# Experiences and Gender Demographics of Researchers in Long‐Term Individual‐Based Study Systems

**DOI:** 10.1002/ece3.74577

**Published:** 2026-11-06

**Authors:** Jillian M. Kusch, Ruth J. Greuel, Chanwoori Jeong, Charlotte E. Regan, Mason R. Stothart, Julie A. Colpitts

**Affiliations:** ^1^ Department of Biology Memorial University of Newfoundland St John's Newfoundland and Labrador Canada; ^2^ Department of Biology University of Saskatchewan Saskatoon Saskatchewan Canada; ^3^ Independent Researcher Calgary Alberta Canada; ^4^ UK Centre for Ecology and Hydrology Penicuik UK; ^5^ Department of Ecosystem and Public Health University of Calgary Calgary Alberta Canada

**Keywords:** bias, demographics, ecology, equity, long‐term research, scholarly publishing

## Abstract

Long‐term individual‐based studies have invaluably advanced our understanding of animal ecology and evolution. Participants involved in the creation and/or maintenance of long‐term study systems benefit from synergistic collaborations, which can provide greater opportunities for co‐authorship. We conducted two surveys to collect anonymous data from current and former participants of long‐term individual‐based studies about leadership structure and authorship patterns as it is unclear whether all participants—regardless of their intersectional identity—derive the same benefits from participation in these systems. Our goal was to collect these data in a less harmful way through self‐reporting gender compared to gendering participants/authors by their first name. We found that gender representation was biased toward men historically and has recently become more equitable across genders. PI gender demographics were positively associated with the gender composition of project participants. Male PIs had higher numbers of publications compared to those of marginalized genders for overall authorship and as last author. Our results parallel similar patterns observed across other science disciplines. This research may serve as a first step in sparking broader reflection, discussion, and action on how gender data is collected and to ensure that the remarkable opportunities offered by long‐term individual‐based study systems are equitably accessible to all.

## Graphical abstract

Participants involved in the creation and/or maintenance of long‐term study systems benefit from synergistic collaborations, which can provide greater opportunities for co‐authorship. We conducted two surveys to collect anonymous data from current and former participants of long‐term individual‐based studies about leadership structure and authorship patterns as it is unclear whether all participants—regardless of their intersectional identity—derive the same benefits from participation in these systems. We found that gender representation was biased toward men historically but has recently become more equitable across genders, and PI gender demographics were positively associated with the gender composition of project participants.

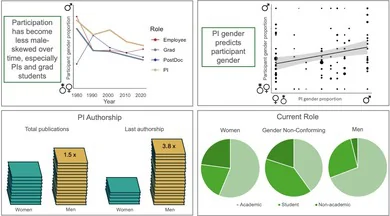

## 1 Introduction

Long‐term individual‐based studies are a cornerstone of ecological research. These studies involve following individual members of a population throughout their lives and typically provide data spanning multiple generations (e.g., the Kluane Red Squirrel Project; the Wytham Woods Great Tit Study). Such individual‐based studies have numerous advantages over their longitudinal population‐scale counterparts which focus on the size and distribution of populations without monitoring individuals. Individual‐based studies can investigate individual variation in vital rates (Rotella 2023), correlations and complexities of life history stages (Richardson and Marshall 2023), fitness costs of reproduction (Festa‐Bianchet et al. 2019), and selection and quantitative genetics (Clutton‐Brock and Sheldon 2010; Festa‐Bianchet et al. 2017). As a result, individual‐based studies have been credited with providing important insights in ecological and evolutionary research (reviewed in: Clutton‐Brock and Sheldon 2010; Sheldon et al. 2022). The scientific advances produced by such studies have frequently translated into high publication rates, with the research produced more commonly appearing in high impact journals and influencing policy than that of their short‐term counterparts (Clutton‐Brock and Sheldon 2010; Hughes et al. 2017).

Given that productivity is often seen as a key factor in a successful scholarly career, the vast output of long‐term individual‐based studies may prove particularly beneficial to the career progression of researchers (Nicholas et al. 2020; Niles et al. 2020). However, for the wider science, technology, engineering, and math (STEM) fields, such benefits may be experienced differently by different segments of the population. STEM fields are traditionally male‐dominated, and despite the increase in women seeking education in these areas, they remain underrepresented in the workforce (Beede et al. 2011; Casad et al. 2021; Sassler et al. 2017). Within ecology, men and women make up approximately equal proportions of undergraduates and young researchers (Damschen et al. 2005), but more senior positions remain dominated by men (Howe‐Walsh and Turnbull 2016; Larivière et al. 2013; Tregenza 2002). As a result, men co‐author more than 70% of articles in ecology (Bradshaw and Courchamp 2018), are more often appointed to editorial boards and as reviewers (Helmer et al. 2017), and there is some evidence that men are cited more often than women authors (Fox and Paine 2019; but see Borsuk et al. 2009; Buckley et al. 2014). Many long‐term individual‐based studies have existed for several decades with some originating as early as the 1940s and 1950s. These studies are likely to have seen pronounced shifts in researcher demographics over their lifespans given that many started under different cultural expectations than those of the present day. Additionally, the deeply entrenched and fieldwork‐focused culture of long‐term ecology studies lends itself to close personal and professional relationships, likely influencing opportunities for inclusion and advancement (Beltran et al. 2020; O'Brien et al. 2020). However, fieldwork has also been demonstrated to be an environment for increased risk of harm for many marginalized individuals (Demery and Pipkin 2020). Consequently, long‐term individual‐based ecology projects provide a unique opportunity to understand if and how gender bias in both project membership and productivity manifest and change over time.

An approximation of the data necessary to answer these questions could conceivably be obtained through literature searches to calculate gender proportions of project membership and manuscript authorship. However, the methods to obtain those proportions are problematic as they rely on categorizing authors based on perceived gender of their first names or making assumptions based on photographs. In particular, naming algorithms produce a binary result—man or woman—ignoring individuals that do not fit within the binary gendering scheme (Rasmussen et al. 2023). These algorithms have also been trained on Anglo‐European names and are more likely to misgender individuals from other cultures (Lockhart et al. 2023). These methods frequently ignore or diminish the experiences of individuals of marginalized genders, furthering the marginalization of those individuals (McLemore 2015). In a recent survey of demographics of LGBTQA‐identified individuals in STEM fields, 7% of respondents self‐identified as transgender, 4% as androgynous, and 11% as genderqueer (Yoder and Mattheis 2016). To our knowledge, no studies within ecology have been conducted where gender was not assigned with either naming algorithms or by excluding gender diverse individuals upon collection of gender diverse data (Borsuk et al. 2009; Budden et al. 2008; Campbell et al. 2013; Fox et al. 2019; Fox and Paine 2019; Maas et al. 2021; Manlove and Belou 2018; Salerno et al. 2019). Studying gender biases carries an ethical responsibility to be inclusive to all genders, especially given historical and ongoing discrimination to marginalized members of the STEM community.

The goal for our study was to assess gender demographics of researchers in long term ecology studies and the potential implications of this for temporal patterns in gender representation across career levels and manuscript authorship using ethical assessments of gender. Following guidelines outlined by Rasmussen et al. (2023), we created surveys for participants to self‐report their gender and their participation in long term ecological projects. Our specific objectives were to:

1. Assess the use of a survey to gather gender data from ecology researchers as an alternate option from assigning gender with naming schemes;
2. Determine trends of gender proportions of researchers of long‐term ecological projects over time and within different roles (e.g., principal investigators, postdoctoral fellows, and graduate students);
3. Investigate whether the relative gender demographics of graduate students and postdoctoral fellows correlate with those of principal investigators; and
4. Determine if authorship differed across genders when considering total authorship as well as author position (first and last).

We predicted that the proportion of men would decrease over time as women and gender nonconforming individuals became more prevalent in the discipline. We also expected that the genders of principal investigators would correlate positively with the genders of their project contributors. Lastly, we predicted that authorship would be lower for women and gender nonconforming individuals compared to men. Within our surveys, we also provided opportunities for respondents to volunteer free‐form responses on what they felt was relevant to their experience working on a long‐term individual‐based study. Our goal was to provide baseline data on demographic patterns among current pillars of long‐term ecological research to spark discussion and action for improving diversity, equity, inclusion, and access in extant and future long‐term individual‐based animal studies.

## 2 Materials and Methods

### 2.1 Positionality Statement

The authors of this paper include cis men, cis women, and gender nonconforming individuals. We have written this paper from the stance that trans women are women, trans men are men, and gender identity and expression exist on a continuum that includes a range of identities. At the time of idea origination, 5 of 6 authors were graduate students, and all authors have personal experience working on one or more long‐term individual‐based ecology projects.

### 2.2 Data Collection

We defined long‐term individual‐based projects as projects that have been running for at least 10 years and where data collection follows individuals across their life histories, rather than population‐level trends. We compiled a list of projects based on prior author knowledge, internet searches, and a literature search. For our literature search, we used the terms “longterm”, “long‐term”, “long term”, “ecology”, “wildlife”, and “individual‐based” to find projects in Google Scholar. Our final list consisted of 58 projects. Our efforts to identify long‐term projects matching our inclusion criteria are unlikely to have been exhaustive (studies rarely sample an entire population), but we did obtain a representative dataset of sufficient size to accomplish our study objectives. Given the possibility that we missed projects fitting our criteria, we also allowed studies to be added during the data collection period as they became known (see Supporting Information for survey details). Eleven respondents added projects that we did not include in the initial list. Our study was based on surveys that were distributed globally. The global distribution of projects was from 6 continents: Africa (*n* = 19), Asia (*n* = 4), Europe (*n* = 17), North America (*n* = 22), Oceania (*n* = 4), and South America (*n* = 3). The geographical distribution of the authorship team of this paper is represented primarily by North American and European regions, which reflects some of the major regions where survey participants and projects are located, and may have been a factor in the distribution of responses we received. Most projects studied birds (*n* = 20) and mammals (*n* = 47), with 1 project focused on amphibians and 1 on reptiles.

### 2.3 Long‐Term Study Membership

To characterize the gender composition of studies across their duration, we contacted up to two principal investigators per study to complete a survey (see Supporting Information for survey details). We contacted principal investigators using faculty email addresses (as found on study/faculty websites) when possible, and sent initial email requests in May 2023, with a follow up sent in July 2023. To reach potential participants with unknown identities or contact information, we also circulated recruitment posters on X (formerly Twitter) and through departmental emails from May to August 2023. For each decade that the study had been running, we asked respondents to note, to the best of their ability, the number of principal investigators (PIs), postdoctoral researchers/research fellows (postdocs), graduate students, and other employees (e.g., field staff and lab managers) involved in the study from each of the following four gender identity groupings: man, woman, non‐binary (an umbrella term that includes gender‐fluid and two‐spirit identities), or gender not listed. It was possible that a PI may have counted an individual more than once per decade if they fulfilled several roles within the project. Numbers were provided by decade, starting with the decade in which the project first began. For example, a study that began in the 1980s provided data for 5 decades (1980–1989, 1990–1999, 2000–2009, 2010–2019, 2020–2023). We anonymized responses upon receipt so that results could not be subsequently linked to specific projects.

### 2.4 Long Term Study Participation and Publication Outputs

To quantify the patterns in publication authorship among participants of long‐term individual‐based studies, we produced a second survey. Specifically, we asked individuals to report their gender identity (man, woman, non‐binary, gender fluid/genderqueer, two‐spirit, transgender, agender, other, or prefer not to specify), their role(s) (PI, postdoc, graduate student, undergraduate student, employee, or volunteer), and the length of time they were involved in one or more long‐term individual‐based studies, as well as the number and type (total authorship, first author, and last author) of publications produced as a result of their involvement in a study/studies. Our surveys also included a free form response section for participants to provide any additional information or experiences they deemed relevant and wanted to share. For gender, we asked individuals to select all that apply as gender can include multiple identities at a time or across time. We circulated this survey via X (formerly Twitter), through departmental emails, and by asking PIs who were contacted regarding the first survey to circulate it. We kept this survey open from May to August 2023. Again, all responses were anonymized in terms of individual and project identity.

All materials used for recruitment and data collection were approved by the University of Saskatchewan Behavioral Research Ethics Board (Beh‐REB ID 1499) before distribution and surveys remained open to responses for 3 months. All authors completed the Tri‐Council Policy Statement: Ethical Conduct for Research Involving Humans Course on Research Ethics (TCPS 2: CORE) before surveys were created and data were collected.

### 2.5 Statistical Analysis

We conducted all analyses in R (version 4.1.2, R Core Team 2024). For the PI survey, we received 45 total responses. We removed 21 surveys that were incomplete, resulting in 24 survey responses for analysis. We received responses for projects that began between 1960 and 2010. We received few responses for studies between 1960 and 1980, so we binned responses from before 1990 (*n* = 14). Responses for each decade were otherwise comparable (1990s: *n* = 11, 2000s: *n* = 17, 2010s: *n* = 18, 2020s: *n* = 18). Across six decades of research, respondents reported 496 men, 654 women, and 12 people of non‐binary or genderqueer identities as participants in long‐term research projects. We calculated the binary proportion of men to women for each role (PI, postdoc, graduate student, employee) in each decade. We then performed a two‐tailed one‐proportion *z*‐test using the ‘binom.test()’ function to determine if each ratio was significantly different (*p* < 0.05) from 1:1.

We then assessed how the proportion of genders changed over time across roles. In an effort to be inclusive, rather than remove the non‐binary and genderqueer individuals, we combined all marginalized genders into one factor for analysis. People of marginalized genders are those that face discriminatory biases due to their gender, which includes cisgender and trans women, and other gender nonconforming individuals. We want to be clear that in no way are gender nonconforming folks a subset of women, and by grouping women and gender nonconforming individuals we are instead looking at the ways that people of all marginalized genders are affected by biases based on their gender. The experiences that each group has are not the same, but the outcomes of exclusion are often similar. We consulted with ten non‐binary colleagues about the ethics of combining genders in this manner. Eight colleagues preferred the combination technique, while two suggested we complete analysis of only men and women in one model and compare it to the model with the combined data. We acknowledge that our consultation sample was not large nor was the feedback unanimous, but we felt it was helpful in determining the most appropriate analysis method. To assess how gender representation changed over time, we bound the number of men and people of marginalized genders as a single object to assess gender proportion as the response variable in a generalized linear mixed model using the ‘glmer()’ function from the ‘lme4’ package (Bates et al. 2015) with a binomial distribution. We included year, role, and their interaction as fixed effects. We included project ID as a random variable to account for differences across projects and repeated sampling across decades.

To assess whether PI gender was associated with the gender composition of project teams, we examined correlations between PI gender and the gender of postdocs, graduate students, and employees across all decades. We performed a generalized linear mixed model as described above. We included the gender of postdocs, graduate students, and employees as the dependent variable, and the PI proportion as the independent variable. We bound male and marginalized genders to be assessed as one dependent variable. We included role, year, the interaction between role and PI proportion, and the interaction between year and PI proportion as fixed effects. We also included project ID as a random effect. We checked for linearity and normality of residuals in all models through diagnostic plots (plotted residuals vs. fitted values and QQ plots). Because experiences of women and other marginalized genders can be diverse, we repeated the above analyses with only men and women as the response variable and included the results in the Supporting Information. However, the results do not differ from the models that include all marginalized genders together and we have therefore chosen to present only the initial model within the text.

For the participant survey, we received 102 total responses. We removed 35 incomplete responses and one duplicate submission. As we gave respondents the option to submit data for each of the long‐term projects they had worked on, 67 completed surveys resulted in 94 data points for analysis. Participants indicated they were non‐binary and/or genderqueer in 7.5% (*n* = 5) of survey responses. We did not receive responses for marginalized genders outside of those listed (e.g., trans or two‐spirit individuals). As with the PI survey, we combined all marginalized genders into one factor for analysis. We received responses from 22 men and 45 people of marginalized genders. Participants were also able to provide the role they filled within the project (PI, postdoc, graduate student, undergraduate student, volunteer, or employee). Due to limited responses in several role categories, we combined undergraduate students, volunteers, and employee responses for increased sample size (*n* = 18). The other possible ‘role’ categories we considered were PI (*n* = 13), postdoctoral fellow (*n* = 16), and graduate student (*n* = 47). We checked if there was a gender bias in recurrent participation in long‐term projects. We determined that men participated in 1.45 ± 0.19 (mean ± standard error) projects on average while those of marginalized genders participated in 1.38 ± 0.10 projects. There was no difference between means using a two‐sided *t*‐test (*t* = −0.35, df = 31.79, *p* = 0.73) suggesting that men and people of marginalized genders were equally likely to participate in multiple long‐term projects. To test for correlations among responses within a participant, we performed one‐way random effects ANOVAs for total authorship, first authorship, and last authorship. After we inspected the data for normality and homogeneity, we log‐transformed the data to achieve normality. We determined that 38%, 46%, and 87% of the variance was explained by within individual differences for total authorship, first, and last authorship, respectively, suggesting that individual responses between projects were not highly correlated. Accordingly, we treated each survey response as an independent sample for further analysis and did not include participant ID as a random effect.

To measure the effect that gender and role had on publication authorship, we performed zero‐inflated hurdle models with the ‘hurdle()’ function (Zeileis et al. 2008) of the ‘pscl’ package (Jackman 2020) with a Poisson distribution for the count model and a binomial distribution for the binomial model. It is likely that whether an individual has authored any publications at all is a separate process from how many an individual has authored if greater than one, likely dependent on role within and/or duration on a project as well as gender. We included the total number of papers that a person authored, the number for which they were first author, and the number for which they were last author as dependent variables. We included gender, role, their interaction, and an offset of the number of years involved on a project as independent variables. We checked for linearity and normality of residuals in all models through diagnostic plots. We also repeated these analyses with a binary men and women response variable, and have included these results in the Supporting Information.

## 3 Results

The gender composition across all roles has generally shifted across decades from being male‐dominated to showing greater representation of marginalized groups (Figure 1). The gender proportion skewed toward men in three instances: PIs (*p* = 0.004) and employees (*p* = 0.008) in the 1960s to 1980s, and PIs (*p* = 0.05) in the 2000s. The gender proportion skewed toward women in later years: employees (*p* = 0.05) in the 2000s, graduate students (*p* < 0.001) and employees (*p* = 0.002) in the 2010s, and graduate students (*p* < 0.001) in the 2020s. Non‐binary individuals were reported within projects starting in 1990.

**FIGURE 1:**
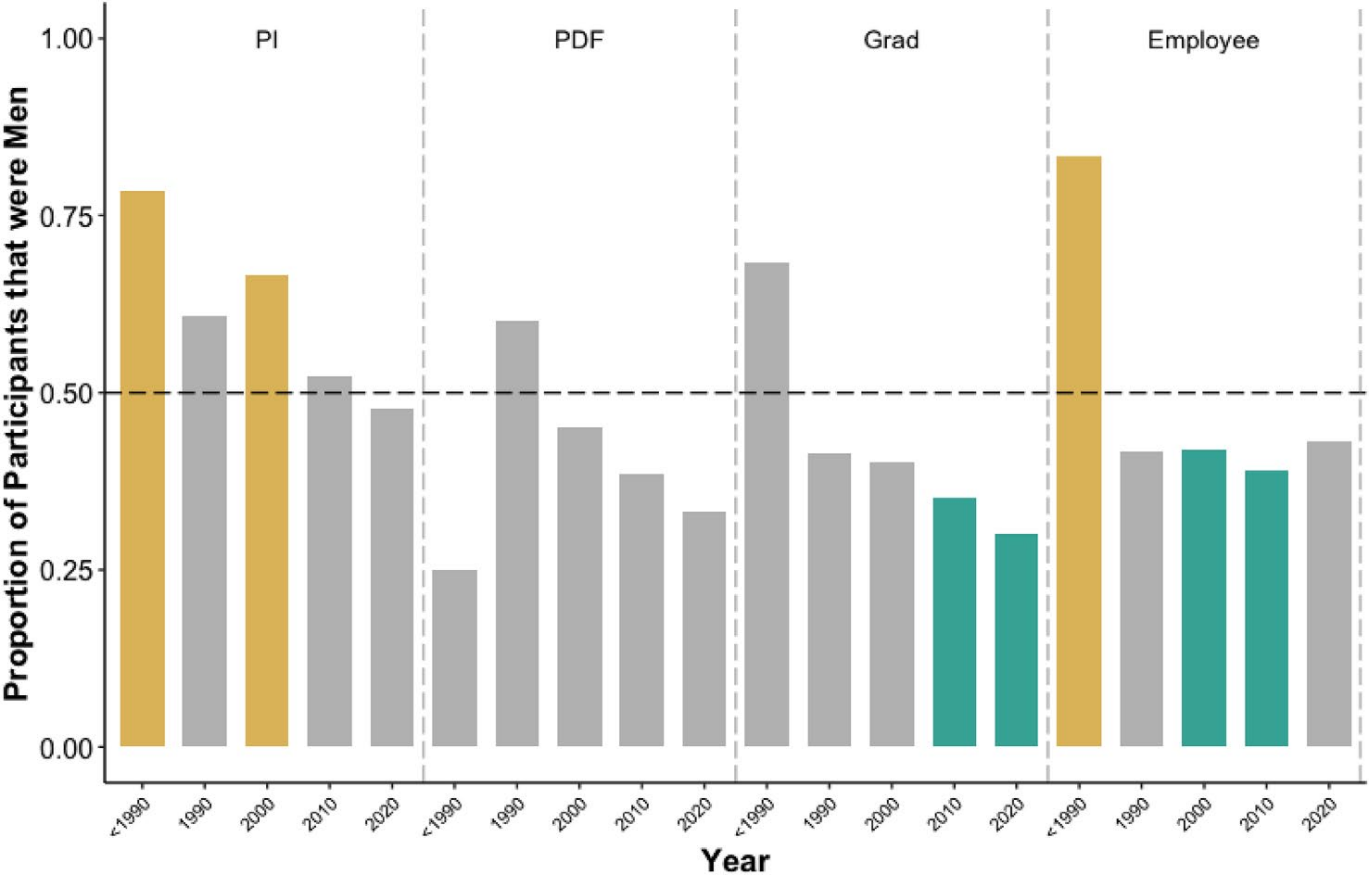
The proportion of men in four roles in long‐term ecological research projects across time. The horizontal black dashed line indicates a proportion of 50% men within each role. Yellow bars indicate that the proportion was significantly skewed toward men. The blue bars indicate that the proportion was significantly skewed toward women. Gray bars indicate that the proportion was not significantly different from 50%.

The results of our model assessing the effect of year and role on the proportion of men and marginalized genders in long‐term ecological research suggest that the proportion of men decreases with time. This effect was most strongly associated for graduate students and PIs (Table 1). However, across all years, men were most likely to be in the role of PI compared to all other roles (Table 1).

**TABLE 1:** Model summary of the effect of role and year on the proportion of men and marginalized genders in long‐term ecology projects. The reference value for role was employee.

Variables	Estimate	*z*	*p*
Intercept	0.20 ± 0.37	0.53	0.60
RoleGrad	0.58 ± 0.52	1.13	0.26
RolePostDoc	0.56 ± 0.85	0.65	0.51
RolePI	1.61 ± 0.57	2.81	< 0.01
Year	−0.11 ± 0.09	−1.21	0.23
RoleGrad:Year	−0.28 ± 0.14	−2.00	0.05
RolePostDoc:Year	−0.21 ± 0.23	−0.91	0.36
RolePI:Year	−0.29 ± 0.16	−1.87	0.06

Broadly, we found that the gender of PIs was a predictor of the gender of other research roles (Figure 2). Graduate students and postdocs of marginalized genders were more likely to be on projects with PIs of marginalized genders, while employees were not affected (Table 2). The tendency for the gender of PIs to predict the gender of other research roles slightly decreased over time (Figure 3). While not significant (*p* = 0.08), the slope of selection across decades decreased from 0.44 before 1990 to near zero by 2000 (−0.01) and remained low through 2020 (0.14). The variance explained by project ID was 0.35 suggesting that 9.8% of the variance was explained by project ID given that the residual variance in a logistic regression is *π* ^2^/3 (Nakagawa and Schielzeth 2010).

**FIGURE 2:**
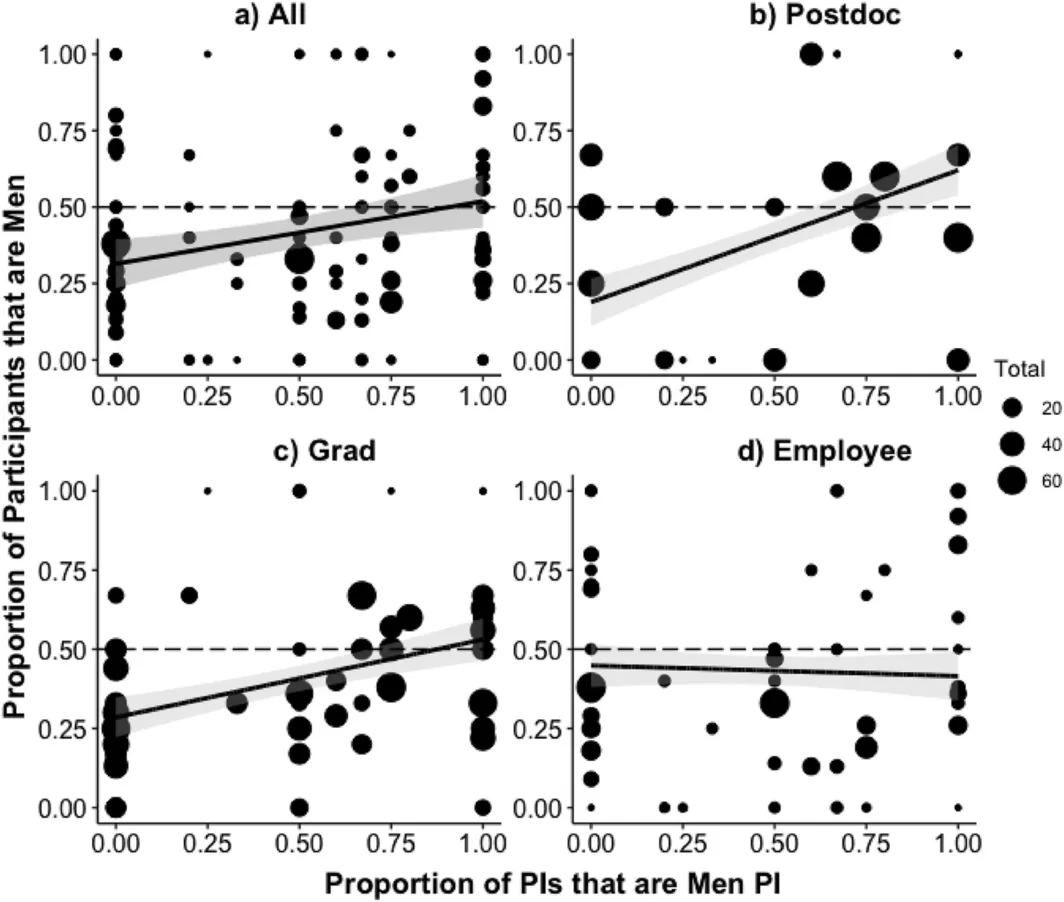
The proportion of genders of PIs plotted against the proportion of genders of hired participants. A value of 1 indicates only men are present, while a value of 0 indicates only marginalized genders are present. The horizontal black dashed line at 0.50 indicates an equal proportion of men to marginalized genders. The gray area represents the 95% confidence interval around the slope. The size of the dot represents the total number of researchers used to calculate each proportion.

**TABLE 2:** Model summary of the effect of PI gender on the gender of participants in long‐term ecology projects. The reference value for role was employee.

Variables	Estimate	*z*	*p*
Intercept	0.12 ± 0.58	0.21	0.84
RoleGrad	−0.97 ± 0.26	−3.71	< 0.01
RolePostDoc	−0.96 ± 0.43	−2.23	0.03
PI_ratio	0.75 ± 0.82	0.91	0.36
Year	−0.05 ± 0.14	−0.35	0.73
RoleGrad:PI_ratio	1.27 ± 0.42	3.01	< 0.01
RolePostDoc:PI_ratio	1.66 ± 0.67	2.46	0.01
Year:PI_ratio	−0.36 ± 0.21	−1.76	0.08

**FIGURE 3:**
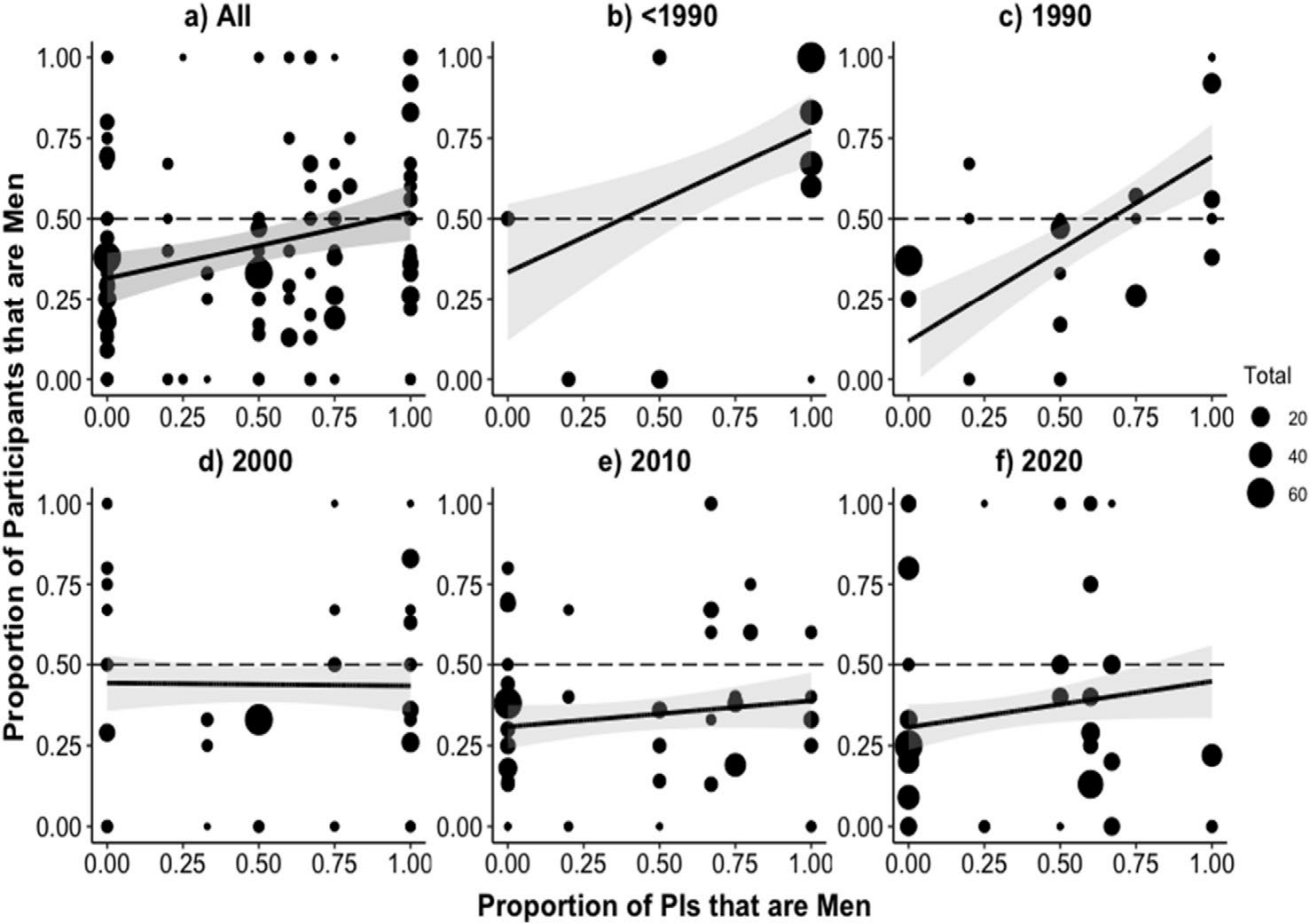
The proportion of genders of PIs plotted against proportion of genders of hired participants across years. Each role within a survey has been plotted separately leading to more data points than survey responses. A value of 1 indicates only men are present, while a value of 0 indicates only marginalized genders are present. The horizontal black dashed line at 0.50 indicates an equal proportion of men to marginalized genders. The gray area represents the 95% confidence interval around the slope. The size of the dot represents the total number of researchers used to calculate each proportion.

We provided a descriptive summary of responses to our participant survey in Table 3. Our data suggest that women remain on projects longer than men or non‐binary peers for all roles other than postdocs where men remain longer. Overall, other than for the role of PI, women appeared to have higher numbers of publications compared to men and non‐binary individuals for the roles where there was non‐binary data. However, these differences disappear when considering length of time on a project and years since participation. When comparing current roles of participants across genders, 3% of men, 23% of women, and 20% of non‐binary participants are not currently working or studying in academia. However, these data were not assessed with statistics due to low sample size and did not include all role types as zero non‐binary PIs participated in our survey.

**TABLE 3:** Summary of results from the participant survey of long‐term individual‐based ecology projects.^a^

Role	Gender	Mean years active	Mean	Mean	Publications			Current role						
			Start	End	Total	Last author	First author	Acad. PI	Non‐Acad. PI	Acad. research	Non‐Acad. research	Non‐Acad. other	Student	Other
PI	Man	15.57	2007	NA	45.57 ± 20.21	31.29 ± 16.35	8.14 ± 5.64	1.00						
PI	Woman	18.17	2004	NA	31.17 ± 11.09	8.17 ± 33.45	9.33 ± 3.70	0.86	0.14					
PDF	Man	5.2	2015	2021	4.89 ± 1.89	0.22 ± 0.22	2.56 ± 1.02	0.33		0.67				
PDF	Woman	4.5	2015	2020	5.5 ± 2.84	0.17 ± 0.17	3.0 ± 1.29	0.17		0.67	0.17			
Grad	Man	3.58	2017	2020	2.0 ± 0.39	0	1.42 ± 0.34			0.36			0.55	0.09
Grad	Woman	4.61	2014	2019	3.39 ± 0.60	0	1.76 ± 0.30			0.46	0.08	0.13	0.33	
Grad	NB	3.5	2017	2020	1.5 ± 0.12	0	1.0 ± 0.25					0.5	0.5	
Other	Man	3.5	2013	2016	1.25 ± 0.95	0	0.75 ± 0.75			0.67			0.33	
Other	Woman	3.92	2012	2016	2.17 ± 1.07	0.08 ± 0.08	0.83 ± 0.37	0.08		0.36	0.09	0.27	0.18	
Other	NB	2	2020	2022	0.5 ± 0.12	0	0.5 ± 0.12			0.5			0.5	

*Note:* For role, other = employees, undergraduates, and volunteers. For gender, NB = non‐binary.

^a^ One non‐binary individual was not included in the table to protect their identity as they were the only data point for their role as a PDF and these summary details could suggest their identity.

Our results for authorship suggest that people of marginalized genders have a decreased number of publications compared to men, but not for all authorship orderings or roles (Figure 4). Using the hurdle models, the zero‐inflated portion that measured the effect of gender on publication in a binary manner (0 publications or at least 1 publication) resulted in nonsignificant results across all authorship models. However, the count portion of the model had significant trends for total authorship and last authorship. When considering role in the number of papers published, the overall number of authored publications among genders was not distinguishable for graduate students (estimate = 0.41 ± 0.26, *p* = 0.12), postdocs (estimate = −0.06 ± 0.35, *p* = 0.85), or undergraduates/volunteers/employees (estimate = 0.05 ± 0.62, *p* = 0.94), but was significant for PIs where PIs of marginalized genders were included on publications at lower numbers than men (odds ratio: 0.39; estimate = −0.95 ± 0.28, *p* < 0.01). For first authorship, a difference in the number of papers published among genders was not distinguishable within roles. For last authorship, only 4 participants (*n* = 1 employee, *n* = 3 postdocs) outside of PIs were listed as the last author so we could not assess for differences between roles, but we did observe a significant correlation where people of marginalized genders published fewer papers than men (odds ratio: 0.23; estimate = −1.47 ± 0.16, *p* < 0.01). We also repeated our analyses with one outlier removed. One male PI had publication numbers far higher than any other datapoint we recorded. However, we observed the same results without this data point (Supporting Information). Full model outputs are available in the Supporting Information.

**FIGURE 4:**
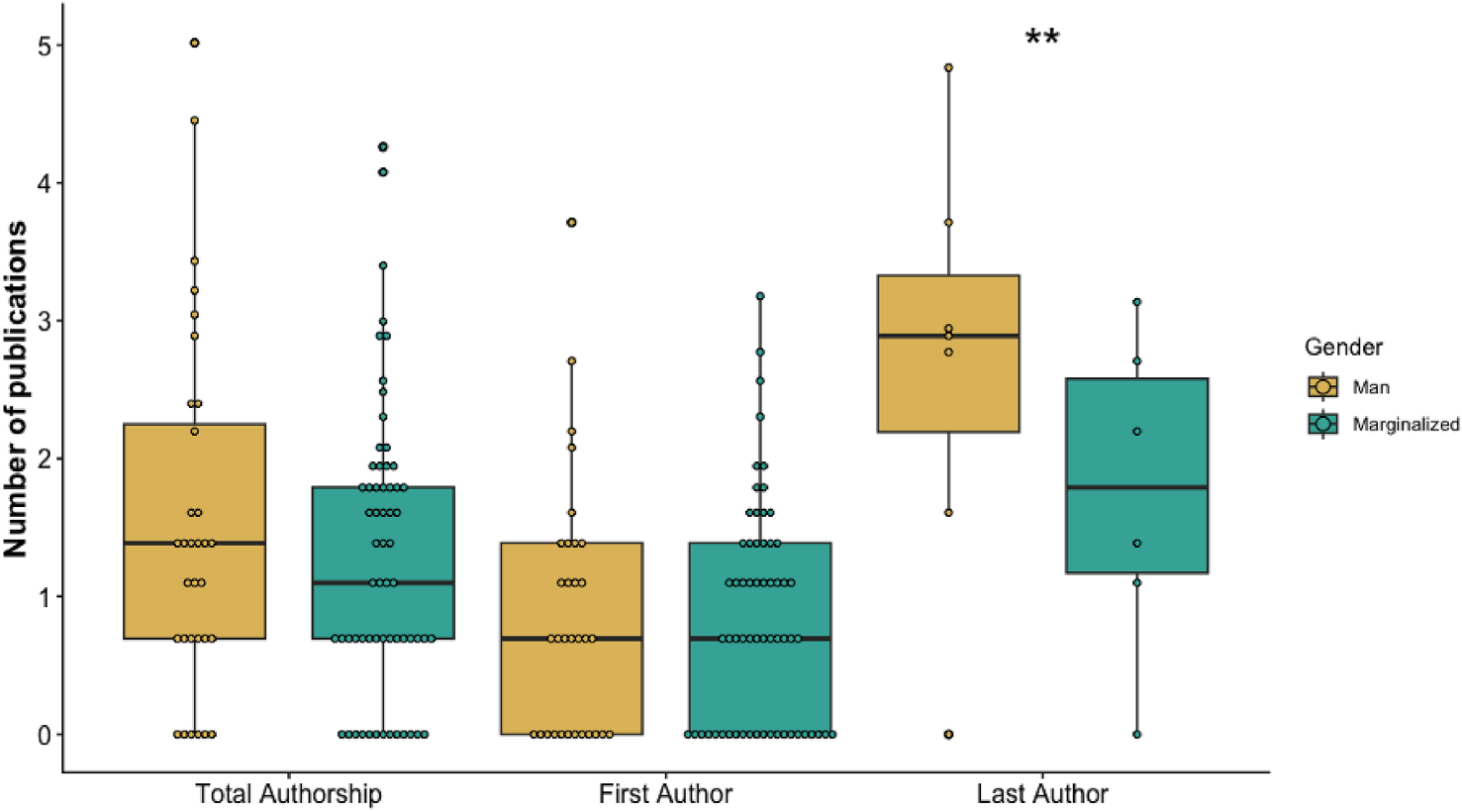
Boxplots of the median number of publications for men and people of marginalized genders across total authorship numbers, first authorship, and last authorship. For last authorship, data has been plotted only for PIs as the difference in publication numbers was driven by PIs. Number of publications has been log‐transformed due to zero‐inflated data for ease of interpretation. **A significant difference between men and people of marginalized genders.

## 4 Discussion

Our study aimed to determine if gender representation across career levels has changed over time and if publication outcomes are equivalent across genders in long‐term individual‐based ecological research projects. We assessed gender demographics through an ethical approach by allowing participants to self‐report their gender. We observed an increase in hiring of people of marginalized genders, particularly as graduate students and PIs. Our results suggest a correlation between the gender of PIs and the gender of participants, where projects with greater representation of marginalized genders among the PI leadership had more graduate students and postdoctoral fellows of marginalized genders. There was considerable variation across projects; however, which suggests that hiring trends are specific to each project. In general, male PIs were more likely to be on publications as a general coauthor and also as last author. Due to small sample size, we advise caution when interpreting these data, but our results do provide evidence that survey methods are a feasible technique for collecting ethical gender data.

The biased gender proportions of hired academics have garnered much interest across STEM fields, and considerable efforts are being made to understand what factors perpetuate this, and how to avoid them (Milgram 2011). Contributing factors include lack of female role models and gender stereotyping (Beede et al. 2011), but not necessarily biased hiring practices (Williams and Ceci 2015). It has previously been suggested that people may use assortative patterns of job selection, where people choose jobs that are historically associated with their gender (Carli et al. 2016; Charles and Bradley 2009; Cheryan et al. 2009). For long‐term ecological projects, people of marginalized genders could be less inclined to seek roles where their own identities are not represented within the PI leadership. Some study systems have historical ties to role models of marginalized genders that has resulted in increased interest in those fields for people of marginalized genders (e.g., women in primatology; Noble 2000). This observation was shared by survey participants in additional comments, which is supported by our results; PI leadership teams that had higher proportions of marginalized genders also had research teams (graduate students, postdocs, and employees) that had higher proportions of marginalized genders. Patterns suggest that assortative recruitment may contribute to observed gender distributions.

We observed a male bias in last authorship, and a similar bias in total authorship. These patterns may reflect the male‐biased PI leadership among projects represented during early decades of the survey data as projects that were underway during these early decades are likely most established and provided the greatest capacity for authorship accumulation. However, the mean number of years of project participation for male PIs was lower than for marginalized PIs (Table 3), suggesting that our results are not due to male‐biased PI proportions from previous decades. Instead, the high participation rate of survey respondents of marginalized genders in long term studies indicates that opportunities for authorship (e.g., invitation to be involved in analysis and writing of research others are leading) may be more commonly offered to male PIs than those of marginalized genders. Previous literature suggests that people of marginalized genders are more likely to be given tasks that do not result in publication or promotion (Babcock et al. 2017; Guarino and Borden 2017). Indeed, survey respondents of marginalized genders mentioned being given administrative tasks over academic work, particularly tasks that did not contribute to manuscript publication such as coordinating equipment and staff for field work or writing summary reports for funding organizations.

In some instances, personal experience reported in the surveys contrasted with our analysis. Some participants reported difficulty including individuals of marginalized genders on manuscripts due to disagreements with PIs about what was considered a valuable contribution. We found no evidence that participants of marginalized genders were underrepresented on manuscripts for all roles other than PIs. These contrasting results might suggest that particular projects are more prone to exclusionary actions than others. While our measure of the effect of gender bias on publications may not have revealed differences for all roles in long‐term projects, the rate at which people of different genders have left academia is variable by gender. Given that these data are descriptive, we do not have the inference as to the mechanism for these departures, but it might suggest that retention issues are still present with long‐term ecology projects as grad students and postdocs of marginalized genders are more likely to leave academia than men. Purposeful and open inclusion on projects could be an important way to support career prospects for people of marginalized genders who participate in long‐term ecological projects.

Some participants referenced intersectional experiences with the written portion of the survey. Intersectionality describes the many identities of a person that contribute to the systemic discrimination they experience which includes gender, race, ethnicity, disability, and sexual orientation (Weldon 2008). Many survey participants mentioned how few Black, Indigenous, and other People of Color (BIPOC) are involved in long‐term ecological projects, especially those located in North America or Europe. Some participants experienced discrimination as researchers of the Global South, especially in publishing, where they received harsher reviews that often focused more on the use of language and grammar over the science being presented. Additionally, participants who self‐disclosed disability were removed from projects despite previous substantial work being completed. While our study focused on gender biases, intersectional identities cannot be divided into categories or studied independently of each other. Marginalization occurs across all visible and invisible minority groups. Our results suggest an intersectional lens should be adopted in future research on the demographic trends and biases observed in patterns of participation within long‐term individual‐based ecological studies.

The use of surveys to collect data often encounters biases such as participation and survivor biases. For the PI survey, recall bias may be present. The recall of participants could be less accurate as project time increases compared to shorter‐length projects. It may also be that people of certain genders were recalled at higher rates than others. We also recognize that PIs may have different experiences with interacting with gender‐diverse individuals, and as a result, may be less likely to report those of gender‐diverse individuals. For example, students may not be “out” to their PIs due to power dynamics or other personal reasons, and therefore additional biases may emerge. The response rate to our participant survey was skewed toward those of marginalized genders with a ratio of 2:1. For anonymity reasons, we did not collect gender data of respondents for the PI survey, but we suggest that we likely had a similar skew as for the participant survey. It has been reported that approximately 50% of undergraduates and 70% of tenure‐track professors in ecology are men (Whelan and Schimel 2019). This discrepancy suggests a sampling bias, which is common when studying gender as people of marginalized genders are more likely to participate in surveys (Becker 2022; Smith 2008). Our results might present a less equitable version of reality as we likely received increased participation from those who experience increased marginalization while those who feel less marginalized were less likely to participate. Alternatively, our data may reflect a more equitable experience than exists if people with little or no publication record were more likely to quit the survey or not participate at all. For example, previous project participants that left academia would have been less likely to receive the survey through our distribution methods. We do not have any evidence to support this suggestion, but survivorship biases may create more optimistic results than would otherwise be presented as those who completed the survey might have had improved experiences than those who did not complete the survey (Walters 2021). Further, the 2020 decade is incomplete so inference may be limited for those data. However, we felt it important to include the 3 years of this decade (at the time of survey responses) as the peak of the COVID‐19 pandemic increased unemployment rates and decreased work hours for women (Collins et al. 2021; Mooi‐Reci and Risman 2021). Our results do not reflect this same effect, given that 2020 data was comparable to 2010 data. However, the impact of the pandemic is an important consideration and may be evident in ways not apparent in our survey results. Given these possible biases, significant care should be taken when generalizing our results to the larger ecology community.

The only ethical and non‐harmful way to study the diversity of gender in science requires self‐reporting of gender identity (Rasmussen et al. 2023). Ethical approaches are increasingly important to serve the entire ecology community as progressively more restrictive legislation is being introduced for trans and other gender nonconforming individuals. Analyses cannot be based on imposition of assigned gender by algorithms or by human assessment. Part of the novelty of our study was asking participants to self‐report gender instead of using naming conventions. However, self‐reporting greatly reduces the amount of data that is collected if people are not inclined to fill out surveys. Our conclusions are limited as we received a small sample size compared to the number of participants that work on long‐term ecological projects. We had to aggregate responses that we would otherwise have preferred to analyze separately (i.e., combining marginalized genders together). One possible solution to ethically accessing gender information is for journals to include voluntary gender identity or pronoun disclosure as a part of manuscript submission to create a catalog of these much‐needed data in a passive way. The increasing frequency with which pronouns are included in people's online profiles and email signatures provides another possible avenue for ethical collection of gender data, if the adoption rate of that practice outweighs the tendency for people to participate in self‐reporting surveys. Alternatively, a standardized reporting network for long‐term ecological research could be established which already exists for other data sharing purposes. The adoption of this practice by allies and senior personnel would do much to normalize self‐reporting for marginalized genders as well. Further, many of the studies that we referenced throughout our paper were limited to binary data of men and women, which is not sufficient for true comparisons or establishment of temporal trends. Researchers who investigate gender demographics must make a concerted effort to include all gender identities in their research to gain a better understanding of how systemic biases and other issues around gender affect academic experiences and productivity. We urge the scientific community to adopt the least harmful means of assigning gender, and prioritize harm reduction over concerns about sample size in order to effectively move the benchmark on such methods.

Long‐term individual‐based ecological projects are essential for gaining a deeper understanding of the natural world. It does appear, however, that some gender‐based differences exist in representation across career stages and number of publications. We suggest that long‐term ecological project leadership conduct self‐assessments of their hiring and publishing practices, look for opportunities to include people of marginalized genders on publications, and consider training to reduce implicit biases. Increasing and retaining diversity requires awareness of and action to counter all types of systemic oppression, and as such we underscore the importance of considering intersectionality of identities for all participants in these long‐term projects. Our research continues to build on well‐established literature that promotes structural change from within academia (Berhe et al. 2022; Hofstra et al. 2020). Our findings serve as a first step in ethically assessing gender demographics to spark broader action and discussion among those who lead and participate in long‐term individual‐based studies and across ecological research.

## Author Contributions

**Jillian M. Kusch:** data curation (supporting), formal analysis (lead), funding acquisition (lead), investigation (equal), methodology (equal), project administration (equal), visualization (lead), writing – original draft (lead), writing – review and editing (lead). **Ruth J. Greuel:** conceptualization (equal), data curation (lead), formal analysis (supporting), methodology (supporting), project administration (supporting), writing – original draft (supporting), writing – review and editing (supporting). **Chanwoori Jeong:** conceptualization (supporting), data curation (supporting), investigation (supporting), methodology (supporting), project administration (supporting), writing – original draft (supporting). **Charlotte E. Regan:** conceptualization (supporting), data curation (supporting), formal analysis (supporting), methodology (supporting), project administration (supporting), writing – original draft (supporting). **Mason R. Stothart:** conceptualization (lead), data curation (supporting), formal analysis (supporting), investigation (supporting), methodology (supporting), project administration (equal), writing – original draft (supporting), writing – review and editing (supporting). **Julie A. Colpitts:** conceptualization (supporting), investigation (supporting), methodology (supporting), project administration (supporting), writing – original draft (supporting), writing – review and editing (supporting).

## Funding

This work was supported by Killam Trusts, Izaak Walton Killam Doctoral Scholarship, Natural Sciences and Engineering Research Council of Canada, Vanier CGS and Alberta Innovates, Graduate Student Scholarship.

## Conflicts of Interest

The authors declare no conflicts of interest.

## Supporting information


**Data S1:** Supporting Information

## Data Availability

Our data is human‐sourced and not available for third‐party use without consent from the survey participants. Human research ethics approval—University of Saskatchewan Behavioral Research Ethics Board (Beh‐REB ID 1499)—requires that these data are not made available. If you are interested in our data, please contact the corresponding author for more information.

## References

[ece374577-bib-0001] Babcock, L. , M. P. Recalde , L. Vesterlund , and L. Weingart . 2017. “Gender Differences in Accepting and Receiving Requests for Tasks With Low Promotability.” American Economic Review 107, no. 3: 714–747. 10.1257/aer.20141734.

[ece374577-bib-0002] Bates, D. , M. Mächler , B. Bolker , and S. Walker . 2015. “Fitting Linear Mixed‐Effects Models Using lme4.” Journal of Statistical Software 67, no. 1: 1–48. 10.18637/jss.v067.i01.

[ece374577-bib-0003] Becker, R. 2022. “Gender and Survey Participation. An Event History Analysis of the Gender Effects of Survey Participation in a Probability‐Based Multi‐Wave Panel Study With a Sequential Mixed‐Mode Design.” Methods, Data, Analyses 16, no. 1: 3–32. 10.12758/mda.2021.08.

[ece374577-bib-0004] Beede, D. , T. Julian , D. Langdon , G. McKittrick , B. Khan , and M. Doms . 2011. “Women in STEM: A Gender Gap to Innovation.” Washington, DC. 10.2139/ssrn.1964782.

[ece374577-bib-0005] Beltran, R. S. , E. Marnocha , A. Race , D. A. Croll , G. H. Dayton , and E. S. Zavaleta . 2020. “Field Courses Narrow Demographic Achievement Gaps in Ecology and Evolutionary Biology.” Ecology and Evolution 10, no. 12: 5184–5196. 10.1002/ece3.6300. PMC7319162 32607142

[ece374577-bib-0006] Berhe, A. A. , R. T. Barnes , M. G. Hastings , et al. 2022. “Scientists From Historically Excluded Groups Face a Hostile Obstacle Course.” Nature 15: 2–4. 10.1038/s41561-021-00868-0.

[ece374577-bib-0007] Borsuk, R. M. , A. E. Budden , R. Leimu , L. W. Aarssen , and C. J. Lortie . 2009. “The Influence of Author Gender, National Language and Number of Authors on Citation Rate in Ecology.” Open Ecology Journal 2, no. 1: 25–28. 10.2174/1874213000902010025.

[ece374577-bib-0008] Bradshaw, C. J. A. , and F. Courchamp . 2018. “Gender Bias When Assessing Recommended Ecology Articles.” Rethinking Ecology 3: 1–12. 10.3897/rethinkingecology.3.24333.

[ece374577-bib-0009] Buckley, H. L. , A. R. Sciligo , K. L. Adair , B. S. Case , and J. M. Monks . 2014. “Is There Gender Bias in Reviewer Selection and Publication Success Rates for the New Zealand Journal of Ecology?” New Zealand Journal of Ecology 38, no. 2: 335–339.

[ece374577-bib-0010] Budden, A. E. , T. Tregenza , L. W. Aarssen , J. Koricheva , R. Leimu , and C. J. Lortie . 2008. “Double‐Blind Review Favours Increased Representation of Female Authors.” Trends in Ecology & Evolution 23, no. 1: 4–6. 10.1016/j.tree.2007.07.008. 17963996

[ece374577-bib-0011] Campbell, L. G. , S. Mehtani , M. E. Dozier , and J. Rinehart . 2013. “Gender‐Heterogeneous Working Groups Produce Higher Quality Science.” PLoS One 8, no. 10: 1–6. 10.1371/journal.pone.0079147. PMC3813606 24205372

[ece374577-bib-0012] Carli, L. L. , L. Alawa , Y. A. Lee , B. Zhao , and E. Kim . 2016. “Stereotypes About Gender and Science: Women ≠ Scientists.” Psychology of Women Quarterly 40, no. 2: 244–260. 10.1177/0361684315622645.

[ece374577-bib-0013] Casad, B. J. , J. E. Franks , C. E. Garasky , et al. 2021. “Gender Inequality in Academia: Problems and Solutions for Women Faculty in STEM.” Journal of Neuroscience Research 99, no. 1: 13–23. 10.1002/jnr.24631. 33103281

[ece374577-bib-0014] Charles, M. , and K. Bradley . 2009. “Indulging Our Gendered Selves? Sex Segregation by Field of Study in 44 Countries.” American Journal of Sociology 114, no. 4: 924–976. 10.1086/595942. 19824299

[ece374577-bib-0015] Cheryan, S. , V. C. Plaut , P. G. Davies , and C. M. Steele . 2009. “Ambient Belonging: How Stereotypical Cues Impact Gender Participation in Computer Science.” Journal of Personality and Social Psychology 97, no. 6: 1045–1060. 10.1037/a0016239. 19968418

[ece374577-bib-0016] Clutton‐Brock, T. , and B. C. Sheldon . 2010. “Individuals and Populations: The Role of Long‐Term, Individual‐Based Studies of Animals in Ecology and Evolutionary Biology.” Trends in Ecology & Evolution 25, no. 10: 562–573. 10.1016/j.tree.2010.08.002. 20828863

[ece374577-bib-0017] Collins, C. , L. C. Landivar , L. Ruppanner , and W. J. Scarborough . 2021. “COVID‐19 and the Gender Gap in Work Hours.” Gender, Work and Organization 28, no. S1: 101–112. 10.1111/gwao.12506. PMC7361447 32837019

[ece374577-bib-0018] Damschen, E. I. , K. M. Rosenfeld , M. Wyer , D. Murphy‐Medley , T. R. Wentworth , and N. M. Haddad . 2005. “Visibility Matters: Increasing Knowledge of Women's Contributions to Ecology.” Frontiers in Ecology and the Environment 3, no. 4: 212–219. 10.1890/1540-9295(2005)003[0212:VMIKOW]2.0.CO;2.

[ece374577-bib-0019] Demery, A.‐J. C. , and A. V. Pipkin . 2020. “Safe Fieldwork Strategies for At‐Risk Individuals, Their Supervisors, and Institutions.” Nature Ecology & Evolution 5: 5–9. 10.1038/s41559-020-01328-5. 33046873

[ece374577-bib-0020] Festa‐Bianchet, M. , S. D. Côté , S. Hamel , and F. Pelletier . 2019. “Long‐Term Studies of Bighorn Sheep and Mountain Goats Reveal Fitness Costs of Reproduction.” Journal of Animal Ecology 88, no. 8: 1118–1133. 10.1111/1365-2656.13002. 31183864

[ece374577-bib-0021] Festa‐Bianchet, M. , M. Douhard , J. M. Gaillard , and F. Pelletier . 2017. “Successes and Challenges of Long‐Term Field Studies of Marked Ungulates.” Journal of Mammalogy 98, no. 3: 612–620. 10.1093/jmammal/gyw227.

[ece374577-bib-0022] Fox, C. W. , M. A. Duffy , D. J. Fairbairn , and J. A. Meyer . 2019. “Gender Diversity of Editorial Boards and Gender Differences in the Peer Review Process at Six Journals of Ecology and Evolution.” Ecology and Evolution 9: 13636–13649. 10.1002/ece3.5794.

[ece374577-bib-0023] Fox, C. W. , and C. E. T. Paine . 2019. “Gender Differences in Peer Review Outcomes and Manuscript Impact at Six Journals of Ecology and Evolution.” Ecology and Evolution 9, no. 6: 3599–3619. 10.1002/ece3.4993. PMC6434606 30962913

[ece374577-bib-0024] Guarino, C. M. , and V. M. H. Borden . 2017. “Faculty Service Loads and Gender: Are Women Taking Care of the Academic Family?” Research in Higher Education 58, no. 6: 672–694. 10.1007/s11162-017-9454-2.

[ece374577-bib-0025] Helmer, M. , M. Schottdorf , A. Neef , and D. Battaglia . 2017. “Gender Bias in Scholarly Peer Review.” eLife 6: 1–18. 10.7554/eLife.21718. PMC5360442 28322725

[ece374577-bib-0026] Hofstra, B. , V. V. Kulkarni , S. M.‐N. Galvez , B. He , D. Jurafsky , and D. A. McFarland . 2020. “The Diversity‐Innovation Pradaox in Science.” Proceedings of the National Academy of Sciences of the United States of America 117, no. 17: 9284–9291. 10.1073/pnas.1915378117. PMC7196824 32291335

[ece374577-bib-0027] Howe‐Walsh, L. , and S. Turnbull . 2016. “Barriers to Women Leaders in Academia: Tales From Science and Technology.” Studies in Higher Education 41, no. 3: 415–428. 10.1080/03075079.2014.929102.

[ece374577-bib-0028] Hughes, B. B. , R. Beas‐Luna , A. K. Barner , et al. 2017. “Long‐Term Studies Contribute Disproportionately to Ecology and Policy.” Bioscience 67, no. 3: 271–278. 10.1093/biosci/biw185.

[ece374577-bib-0029] Jackman, S. 2020. “pscl: Classes and Methods for R Developed in the Political Science Computational Laboratory.” Sydney, New South Wales, Australia.: United States Studies Centre, University of Sydney. https://github.com/atahk/pscl/.

[ece374577-bib-0030] Larivière, V. , C. Ni , Y. Gingras , B. Cronin , and C. R. Sugimoto . 2013. “Global Gender Disparities in Science.” Nature 504, no. 7479: 211–213. 10.1038/504211a 24350369

[ece374577-bib-0031] Lockhart, J. W. , M. M. King , and C. Munsch . 2023. “Name‐Based Demographic Inference and the Unequal Distribution of Misrecognition.” Nature Human Behaviour 7: 1084–1095. 10.1038/s41562-023-01587-9. 37069295

[ece374577-bib-0032] Maas, B. , R. J. Pakeman , L. Godet , L. Smith , V. Devictor , and R. Primack . 2021. “Women and Global South Strikingly Underrepresented Among Top‐Publishing Ecologists.” Conservation Letters 14, no. 4: 1–9. 10.1111/conl.12797.

[ece374577-bib-0033] Manlove, K. R. , and R. M. Belou . 2018. “Authors and Editors Assort on Gender and Geography in High‐Rank Ecological Publications.” PLoS One 13, no. 2: 1–13. 10.1371/journal.pone.0192481. PMC5805316 29420647

[ece374577-bib-0034] McLemore, K. A. 2015. “Experiences With Misgendering: Identity Misclassification of Transgender Spectrum Individuals.” Self and Identity 14, no. 1: 51–74. 10.1080/15298868.2014.950691.

[ece374577-bib-0035] Milgram, D. 2011. “How to Recruit Women and Girls to the Science, Technology, Engineering, and Math (STEM) Classroom.” Technology and Engineering Teacher 71, no. 3: 4–11.

[ece374577-bib-0036] Mooi‐Reci, I. , and B. J. Risman . 2021. “The Gendered Impacts of COVID‐19: Lessons and Reflections.” Gender and Society 35, no. 2: 161–167. 10.1177/08912432211001305.

[ece374577-bib-0037] Nakagawa, S. , and H. Schielzeth . 2010. “Repeatability for Gaussian and Non‐Gaussian Data: A Practical Guide for Biologists.” Biological Reviews 85, no. 4: 935–956. 10.1111/j.1469-185X.2010.00141.x. 20569253

[ece374577-bib-0038] Nicholas, D. , H. R. Jamali , E. Herman , et al. 2020. “A Global Questionnaire Survey of the Scholarly Communication Attitudes and Behaviours of Early Career Researchers.” Learned Publishing 33: 197–210. 10.1002/leap.1286.

[ece374577-bib-0039] Niles, M. T. , L. A. Schimanski , E. C. McKiernan , and J. P. Alperin . 2020. “Why We Publish Where We Do: Faculty Publishing Values and Their Relationship to Review, Promotion and Tenure Expectations.” PLoS One 15, no. 3: 1–15. 10.1371/journal.pone.0228914. PMC7065820 32160238

[ece374577-bib-0040] Noble, B. 2000. “Politics, Gender, and Worldly Primatology: The Goodall‐Fossey Nexus.” In Primate Encounters: Models of Science, Gender, and Society, edited by S. C. Strum and L. M. Fedigan , 436–462. University of Chicago Press.

[ece374577-bib-0041] O'Brien, L. T. , H. L. Bart , and D. M. Garcia . 2020. “Why Are There So Few Ethnic Minorities in Ecology and Evolutionary Biology? Challenges to Inclusion and the Role of Sense of Belonging.” Social Psychology of Education 23, no. 2: 449–477. 10.1007/s11218-019-09538-x.

[ece374577-bib-0042] R Core Team . 2024. R: A Language and Environment for Statistical Computing Austria.

[ece374577-bib-0043] Rasmussen, K. , J. Chen , R. L. Colquhoun , et al. 2023. “Gender Inclusive Methods in Studies of STEM Practitioners.” ArXiv. http://arxiv.org/abs/2307.15802.

[ece374577-bib-0044] Richardson, E. L. , and D. J. Marshall . 2023. “Mapping the Correlations and Gaps in Studies of Complex Life Histories.” Ecology and Evolution 13, no. 2: 1–14. 10.1002/ece3.9809. PMC9937794 36820248

[ece374577-bib-0045] Rotella, J. J. 2023. “Patterns, Sources, and Consequences of Variation in Age‐Specific Vital Rates: Insights From a Long‐Term Study of Weddell Seals.” Journal of Animal Ecology 92, no. 3: 552–567. 10.1111/1365-2656.13870. 36495476

[ece374577-bib-0046] Salerno, P. E. , M. Páez‐Vacas , J. M. Guayasamin , and J. L. Stynoski . 2019. “Male Principal Investigators (Almost) Don't Publish With Women in Ecology and Zoology.” PLoS One 14, no. 6: 1–14. 10.1371/journal.pone.0218598. PMC6583967 31216351

[ece374577-bib-0047] Sassler, S. , J. Glass , Y. Levitte , and K. M. Michelmore . 2017. “The Missing Women in STEM? Assessing Gender Differentials in the Factors Associated With Transition to First Jobs.” Social Science Research 63: 192–208. 10.1016/j.ssresearch.2016.09.014. PMC5315093 28202143

[ece374577-bib-0048] Sheldon, B. C. , L. E. B. Kruuk , and S. C. Alberts . 2022. “The Expanding Value of Long‐Term Studies of Individuals in the Wild.” Nature Ecology and Evolution 6, no. 12: 1799–1801. 10.1038/s41559-022-01940-7. 36348080

[ece374577-bib-0049] Smith, W. G. 2008. “Does Gender Influence Online Survey Participation? A Record‐Linkage Analysis of University Faculty Online Survey Response Behavior.” San José, CA. http://files.eric.ed.gov/fulltext/ED501717.pdf.

[ece374577-bib-0050] Tregenza, T. 2002. “Gender Bias in the Refereeing Process?” Trends in Ecology & Evolution 17, no. 8: 349–350.

[ece374577-bib-0051] Walters, W. H. 2021. “Survey Design, Sampling, and Significance Testing: Key Issues.” Journal of Academic Librarianship 47, no. 3: 102344. 10.1016/j.acalib.2021.102344.

[ece374577-bib-0052] Weldon, S. L. 2008. “Intersectionality.” In Politics, Gender, and Concepts, edited by G. Goertz and A. G. Mazur , 193–218. Cambridge University Press. 10.1017/cbo9780511755910.

[ece374577-bib-0053] Whelan, A. M. , and D. S. Schimel . 2019. “Authorship and Gender in ESA Journals.” Bulletin of the Ecological Society of America 100, no. 3: 1–9. 10.1002/bes2.1567.

[ece374577-bib-0054] Williams, W. M. , and S. J. Ceci . 2015. “National Hiring Experiments Reveal 2:1 Faculty Preference for Women on STEM Tenure Track.” Proceedings of the National Academy of Sciences of the United States of America 112, no. 17: 5360–5365. 10.1073/pnas.1418878112. PMC4418903 25870272

[ece374577-bib-0055] Yoder, J. B. , and A. Mattheis . 2016. “Queer in STEM: Workplace Experiences Reported in a National Survey of LGBTQA Individuals in Science, Technology, Engineering, and Mathematics Careers.” Journal of Homosexuality 63, no. 1: 1–27. 10.1080/00918369.2015.1078632. 26241115

[ece374577-bib-0056] Zeileis, A. , C. Kleiber , and S. Jackman . 2008. “Regression Models for Count Data in R.” Journal of Statistical Software 27, no. 8: 1–25.

