# Supplementary material for: Experiences and Gender Demographics of Researchers in Long‐Term Individual‐Based Study Systems
Source: Ecol Evol. 2026 Nov 6;16(11):e74577. doi: 10.1002/ece3.74577 (PMC13743406; doi:10.1002/ece3.74577)
Supplement: Supplementary file 1 [file ECE3-16-e74577-s001.zip › ece374577-sup-0001-supinfo01.pdf]

UNIVERSITY OF SASKATCHEWAN

Copy of Researcher gender in long-term individual-based ecological studies [Participant Survey]

Researcher gender in long-term individual-based ecological studies [Participants]

You are invited to participate in a web-based online survey on the membership and publication breakdown of long-term individual-based ecology studies over time. This is a research project being conducted by a small group of early-career biologists. It should take approximately 10 minutes to complete.

PARTICIPATION
Your participation in this survey is voluntary. You may refuse to take part in the research or exit the survey at any time without penalty. You are free to decline to answer any question for any reason, and your consent may be withdrawn at any point. Simply exit your browser without completing the survey if you no longer wish to participate. Since the survey is anonymous, once it is submitted it cannot be removed.

BENEFITS
You will receive no direct benefits from participating in this research study. However, your responses will help further the knowledge surrounding gender demographics of researchers in STEM, specifically in a highly important and productive segment of ecological research. Identifying biases, or lack thereof, may help us understand barriers to equality and improve the wellbeing of researchers and the projects they participate in over time.

RISKS

The possible risks of the study are minimal. You may experience some discomfort and/or frustration answering repetitive survey questions that could identify patterns in hiring practices or record keeping, for example.

CONFIDENTIALITY
Your data will be stored in a password protected electronic format. Although you will be asked to provide some personal information, no identifying information such as your name, email address, or IP address will be collected. Only the name(s) of the project(s) for which you are responding will be required, which will later be coded to ensure anonymity. No one will be able to identify you or your answers, and no one will know whether or not you participated in the study. All data will be published in aggregate form, and no project(s) or persons will be singled out or specifically identified in the dissemination of results. In rare cases, it may be possible for your identity to be surmised based on your project involvement. However, identification of individuals and projects will not be possible in the final compiled dataset, nor will anyone or their project(s) be identified in the presentation of results. Because the

named PI may have an academic relationship with some of you, they will not have access to the data until it has been anonymized and the project names removed. This survey is hosted by Survey Monkey, and the data will be stored in facilities hosted in Canada. To see Survey Monkey's privacy policy, please click here: https://www.surveymonkey.com/mp/legal/privacy-policy/

FOLLOW UP

The results of this survey will form the basis of a manuscript to be submitted to a scientific journal. If you wish to view a summary of the results or receive a copy of the submitted paper, please contact the research team via email (click Next).

UNIVERSITY OF SASKATCHEWAN

Copy of Researcher gender in long-term individual-based ecological studies [Participant Survey]

CONTACT
If you have questions or concerns at any time, please don't hesitate to contact the research team

ETHICS APPROVAL

This research project has been approved on ethical grounds by the University of Saskatchewan Behavioural Research Ethics Board. Any questions regarding your rights as a participant may be addressed to that committee through the Research Ethics Office: ethics.office@usask.ca; (306) 966-2975; out of town participants may call toll free (888) 966-2975.

ELECTRONIC CONSENT: Please indicate your consent below. If you do not wish to participate in this study, you may close your browser window now. You may print a copy of this consent form for your records. By clicking "Agree" below, you are confirming that:

- **You have read the above information**
- **You voluntarily agree to participate**
- **You are 18 years of age or older**

* 1. I consent to participate in this survey

- ◯ Agree
- ◯ Disagree

UNIVERSITY OF SASKATCHEWAN

Copy of Researcher gender in long-term individual-based ecological studies [Participant Survey]

Gender self-reporting

In answering the following questions, please answer as accurately as possible. If an exact answer is not known, please provide your best estimate.

2. What is your gender (please select all that apply):

- ☐ Man
- ☐ Woman
- ☐ Non-binary
- ☐ Gender-Fluid or Genderqueer
- ☐ My gender is not listed (please specify)
- ☐ Two-Spirit
- ☐ Transgender
- ☐ Agender
- ☐ Prefer not to specify

UNIVERSITY OF SASKATCHEWAN

Copy of Researcher gender in long-term individual-based ecological studies [Participant Survey]

Current employment

3. In which category does your current employment best fit? Please select from the dropdown menu:

UNIVERSITY OF SASKATCHEWAN

Copy of Researcher gender in long-term individual-based ecological studies

[Participant Survey]

Long-term individual-based research projects

4. In which long-term individual-based study were you involved? Please select from the dropdown menu. If you have been involved in more than one long-term individual based study, you will be given the opportunity to provide responses for those studies as well. Please indicate only one at this time.

University of Saskatchewan

Copy of Researcher gender in long-term individual-based ecological studies [Participant Survey]

Participation

5. For the project you indicated above, in what capacity were you involved? Please select all that apply:

- [ ] Undergraduate Student
- [ ] Graduate Student
- [ ] Postdoctoral Fellow
- [ ] Principal Investigator
- [ ] Employee
- [ ] Volunteer

University of Saskatchewan

Copy of Researcher gender in long-term individual-based ecological studies [Participant Survey]

Timeline

Please provide numeric responses for the following questions:

6. In what year did you begin work on this project?

7. In what year did you cease work on this project? If your participation is ongoing, please enter NA.

UNIVERSITY OF SASKATCHEWAN

Copy of Researcher gender in long-term individual-based ecological studies [Participant Survey]

Publication history

Please provide numeric responses to the following questions

8. How many publications have you (co-)authored in association with the project indicated above?

9. Of the above number of publications, on how many were you last author?

10. Of the above number of publications, on how many were you first author?

UNIVERSITY OF SASKATCHEWAN

Copy of Researcher gender in long-term individual-based ecological studies [Participant Survey]

Continue?

* 11. Are there any other projects for which you would like to provide responses?

- ◯ Yes
- ◯ No

UNIVERSITY OF SASKATCHEWAN

Copy of Researcher gender in long-term individual-based ecological studies [Participant Survey]

Copy of page: Long-term individual-based research projects

12. In which long-term individual-based study were you involved? Please select from the dropdown menu. If you have been involved in more than one long-term individual based study, you will be given the opportunity to provide responses for those studies as well. Please indicate only one at this time.

UNIVERSITY OF SASKATCHEWAN

Copy of Researcher gender in long-term individual-based ecological studies [Participant Survey]

Copy of page: Participation

13. For the project you indicated above, in what capacity were you involved? Please select all that apply:

- Undergraduate Student
- Graduate Student
- Postdoctoral Fellow
- Principal Investigator
- Employee
- Volunteer

UNIVERSITY OF SASKATCHEWAN

Copy of Researcher gender in long-term individual-based ecological studies [Participant Survey]

Copy of page: Timeline

Please provide numeric responses for the following questions:

14. In what year did you begin work on this project?

15. In what year did you cease work on this project? If your participation is ongoing, please enter NA.

UNIVERSITY OF SASKATCHEWAN

Copy of Researcher gender in long-term individual-based ecological studies [Participant Survey]

Copy of page: Publication history

Please provide numeric responses to the following questions

16. How many publications have you (co-)authored in association with the project indicated above?

17. Of the above number of publications, on how many were you last author?

18. Of the above number of publications, on how many were you first author?

UNIVERSITY OF SASKATCHEWAN

Copy of Researcher gender in long-term individual-based ecological studies [Participant Survey]

Copy of page: Continue?

* 19. Are there any other projects for which you would like to provide responses?

- ◯ Yes
- ◯ No

UNIVERSITY OF SASKATCHEWAN

Copy of Researcher gender in long-term individual-based ecological studies [Participant Survey]

Copy of page: Long-term individual-based research projects

20. In which long-term individual-based study were you involved? Please select from the dropdown menu. If you have been involved in more than one long-term individual based study, you will be given the opportunity to provide responses for those studies as well. Please indicate only one at this time.

UNIVERSITY OF SASKATCHEWAN

Copy of Researcher gender in long-term individual-based ecological studies [Participant Survey]

Copy of page: Participation

21. For the project you indicated above, in what capacity were you involved? Please select all that apply:

- [ ] Undergraduate Student
- [ ] Graduate Student
- [ ] Postdoctoral Fellow
- [ ] Principal Investigator
- [ ] Employee
- [ ] Volunteer

UNIVERSITY OF SASKATCHEWAN

Copy of Researcher gender in long-term individual-based ecological studies [Participant Survey]

Copy of page: Timeline

Please provide numeric responses for the following questions:

22. In what year did you begin work on this project?

23. In what year did you cease work on this project? If your participation is ongoing, please enter NA.

UNIVERSITY OF SASKATCHEWAN

Copy of Researcher gender in long-term individual-based ecological studies [Participant Survey]

Copy of page: Publication history

Please provide numeric responses to the following questions

24. How many publications have you (co-)authored in association with the project indicated above?

25. Of the above number of publications, on how many were you last author?

26. Of the above number of publications, on how many were you first author?

UNIVERSITY OF SASKATCHEWAN

Copy of Researcher gender in long-term individual-based ecological studies [Participant Survey]

Copy of page: Continue?

* 27. Are there any other projects for which you would like to provide responses?

- Yes
- No

UNIVERSITY OF SASKATCHEWAN

Copy of Researcher gender in long-term individual-based ecological studies [Participant Survey]

Copy of page: Long-term individual-based research projects

28. In which long-term individual-based study were you involved? Please select from the dropdown menu. If you have been involved in more than one long-term individual based study, you will be given the opportunity to provide responses for those studies as well. Please indicate only one at this time.

UNIVERSITY OF SASKATCHEWAN

Copy of Researcher gender in long-term individual-based ecological studies [Participant Survey]

Copy of page: Participation

29. For the project you indicated above, in what capacity were you involved? Please select all that apply:

- [ ] Undergraduate Student
- [ ] Graduate Student
- [ ] Postdoctoral Fellow
- [ ] Principal Investigator
- [ ] Employee
- [ ] Volunteer

UNIVERSITY OF SASKATCHEWAN

Copy of Researcher gender in long-term individual-based ecological studies [Participant Survey]

Copy of page: Timeline

Please provide numeric responses for the following questions:

30. In what year did you begin work on this project?

31. In what year did you cease work on this project? If your participation is ongoing, please enter NA.

UNIVERSITY OF SASKATCHEWAN

Copy of Researcher gender in long-term individual-based ecological studies [Participant Survey]

Copy of page: Publication history

Please provide numeric responses to the following questions

32. How many publications have you (co-)authored in association with the project indicated above?

33. Of the above number of publications, on how many were you last author?

34. Of the above number of publications, on how many were you first author?

UNIVERSITY OF SASKATCHEWAN

Copy of Researcher gender in long-term individual-based ecological studies [Participant Survey]

Copy of page: Continue?

* 35. Are there any other projects for which you would like to provide responses?

- Yes
- No

UNIVERSITY OF SASKATCHEWAN

Copy of Researcher gender in long-term individual-based ecological studies [Participant Survey]

Copy of page: Long-term individual-based research projects

36. In which long-term individual-based study were you involved? Please select from the dropdown menu. If you have been involved in more than one long-term individual based study, you will be given the opportunity to provide responses for those studies as well. Please indicate only one at this time.

University of Saskatchewan

Copy of Researcher gender in long-term individual-based ecological studies [Participant Survey]

Copy of page: Participation

37. For the project you indicated above, in what capacity were you involved? Please select all that apply:

- [ ] Undergraduate Student
- [ ] Graduate Student
- [ ] Postdoctoral Fellow
- [ ] Principal Investigator
- [ ] Employee
- [ ] Volunteer

University of Saskatchewan

Copy of Researcher gender in long-term individual-based ecological studies [Participant Survey]

Copy of page: Timeline

Please provide numeric responses for the following questions:

38. In what year did you begin work on this project?

39. In what year did you cease work on this project? If your participation is ongoing, please enter NA.

University of Saskatchewan

Copy of Researcher gender in long-term individual-based ecological studies [Participant Survey]

Copy of page: Publication history

Please provide numeric responses to the following questions

40. How many publications have you (co-)authored in association with the project indicated above?

41. Of the above number of publications, on how many were you last author?

42. Of the above number of publications, on how many were you first author?

University of Saskatchewan

Copy of Researcher gender in long-term individual-based ecological studies [Participant Survey]

Final thoughts

43. Is there any other information you wish to provide? Please note that all answers will remain anonymous and no direct quotes will be used.
