# Supplementary material for: Experiences and Gender Demographics of Researchers in Long‐Term Individual‐Based Study Systems
Source: Ecol Evol. 2026 Nov 6;16(11):e74577. doi: 10.1002/ece3.74577 (PMC13743406; doi:10.1002/ece3.74577)
Supplement: Supplementary file 1 [file ECE3-16-e74577-s001.zip › ece374577-sup-0002-supinfo02.pdf]

UNIVERSITY OF SASKATCHEWAN

Copy of Researcher gender in long-term individual-based ecological studies [Principal Investigator Survey]

Researcher gender in long-term individual-based ecological studies

As a Principal Investigator or long standing member of a long-term individual-based project, you are invited to participate in a web-based online survey on the membership of long-term individual-based ecology studies over time. This is a research project being conducted by a small group of early-career biologists. It should take approximately 10 minutes to complete.

PARTICIPATION
Your participation in this survey is voluntary. You may refuse to take part in the research or exit the survey at any time without penalty. You are free to decline to answer any question for any reason, and your consent may be withdrawn at any point. Simply exit your browser without completing the survey if you no longer wish to participate. Since the survey is anonymous, once it is submitted it cannot be removed.

BENEFITS
You will receive no direct benefits from participating in this research study. However, your responses will help further the knowledge surrounding gender demographics of researchers in STEM, specifically in a highly important and productive segment of ecological research. Identifying biases, or lack thereof, may help us understand barriers to equality and improve the wellbeing of researchers and the projects they participate in over time.

RISKS
The possible risks of the study are minimal. You may experience some discomfort and/or frustration answering repetitive survey questions that could identify patterns in hiring practices or record keeping, for example.

CONFIDENTIALITY
Your data will be stored in a password protected electronic format. No identifying information such as your name, email address, or IP address will be collected. Only the name of the project for which you are responding will be required, which will later be coded to ensure anonymity. No one will be able to identify you or your answers, and no one will know whether or not you participated in the study. All data will be published in aggregate form, and no project(s) will be singled out or specifically identified in the dissemination of results. In the rare case that only one person has been contacted for a given project, it may be possible for your identity to be surmised based on the project for which you answer. However, identification of individuals and projects will not be possible in the final compiled dataset, nor will anyone or their project(s) be identified in the presentation of results. Because the named PI may have an academic relationship with some of you, they will not have

access to the data until it has been anonymized and the project names removed. This survey is hosted by Survey Monkey, and the data will be stored in facilities hosted in Canada. To see Survey Monkey's privacy policy, please click here: https://www.surveymonkey.com/mp/legal/privacy-policy/

FOLLOW UP
The results of this survey will form the basis of a manuscript to be submitted to a scientific journal. If you wish to view a summary of the results or receive a copy of the submitted paper, please contact the research team via email (click Next).

UNIVERSITY OF SASKATCHEWAN

Copy of Researcher gender in long-term individual-based ecological studies [Principal Investigator Survey]

CONTACT
If you have questions or concerns at any time, please don't hesitate to contact the research team

ETHICS APPROVAL

This research project has been approved on ethical grounds by the University of Saskatchewan Behavioural Research Ethics Board. Any questions regarding your rights as a participant may be addressed to that committee through the Research Ethics Office: ethics.office@usask.ca; (306) 966-2975; out of town participants may call toll free (888) 966-2975.

ELECTRONIC CONSENT: Please indicate your consent below. If you do not wish to participate in this study, you may close your browser window now. You may print a copy of this consent form for your records. By clicking "Agree" below, you are confirming that:

- **You have read the above information**
- **You voluntarily agree to participate**
- **You are 18 years of age or older**

* 1. I consent to participate in this survey

◯ Agree

◯ Disagree

UNIVERSITY OF SASKATCHEWAN

Copy of Researcher gender in long-term individual-based ecological studies [Principal Investigator Survey]

Welcome and thank you for participating in this survey.

In answering the following questions, please answer as accurately as possible. If an exact answer is not known, please provide your best estimate. If any of the participants in your project are known to you as transgender, please include them with the category that most closely resembles how they have identified themselves to you.

2. For which long term individual-based study are you providing responses? Please select from the dropdown menu.

3. In which decade did the study begin?

- ○ 1950-1959
- ○ 1960-1969
- ○ 1970-1979
- ○ 1980-1989
- ○ 1990-1999
- ○ 2000-2009
- ○ 2010-2019
- ○ Since 2020

UNIVERSITY OF SASKATCHEWAN

Copy of Researcher gender in long-term individual-based ecological studies [Principal Investigator Survey]

1950s

4. In the 1950s how many Principal Investigators of each gender were involved in the project? A Principal Investigator on the project is defined as someone who actively participates in at least two of the following activities: seeking and administering core project funding, supervising student and/or postdoctoral projects, finalizing/approving hiring decisions, participates in project design, and/or holds a faculty position at a research institution.

Man	
Woman	
Non-Binary, Gender-Fluid and/or Two-Spirit	
Gender not listed	

5. In the 1950s how many Postdoctoral Researchers or Research Fellows of each gender were involved in the project? A Postdoctoral Researcher/Research Fellow on the project is defined as someone who has completed their PhD and has been hired by a Principal Investigator to conduct research pertaining to the project with a fixed end date.

Man	
Woman	
Non-Binary, Gender-Fluid and/or Two-Spirit	
Gender not listed	

6. In the 1950s how many graduate students of each gender were involved in the project? A graduate student on the project is defined as someone who is conducting their research as part of a graduate degree (i.e. Master’s or Doctorate).

Man	
Woman	
Non-Binary, Gender-Fluid and/or Two-Spirit	
Gender not listed	

7. In the 1950s how many other employees (such as field technicians and lab managers) of each gender were involved in the project? An employee of the project is defined as someone who holds a paid position to actively participate in project research, but does not fit into any of the above categories.

Man	
Woman	
Non-Binary, Gender-Fluid and/or Two-Spirit	
Gender not listed	

UNIVERSITY OF SASKATCHEWAN

Copy of Researcher gender in long-term individual-based ecological studies [Principal Investigator Survey]

1960s

8. In the 1960s how many Principal Investigators of each gender were involved in the project? A Principal Investigator on the project is defined as someone who actively participates in at least two of the following activities: seeking and administering core project funding, supervising student and/or postdoctoral projects, finalizing/approving hiring decisions, participates in project design, and/or holds a faculty position at a research institution.

Man	
Woman	
Non-Binary, Gender-Fluid and/or Two-Spirit	
Gender not listed	

9. In the 1960s how many Postdoctoral Researchers or Research Fellows of each gender were involved in the project? A Postdoctoral Researcher/Research Fellow on the project is defined as someone who has completed their PhD and has been hired by a Principal Investigator to conduct research pertaining to the project with a fixed end date.

Man

Woman

Non-Binary, Gender-Fluid and/or Two-Spirit

Gender not listed

10. In the 1960s how many graduate students of each gender were involved in the project? A graduate student on the project is defined as someone who is conducting their research as part of a graduate degree (i.e. Master's or Doctorate).

Man

Woman

Non-Binary, Gender-Fluid and/or Two-Spirit

Gender not listed

11. In the 1960s how many other employees (such as field technicians and lab managers) of each gender were involved in the project? An employee of the project is defined as someone who holds a paid position to actively participate in project research, but does not fit into any of the above categories.

Man

Woman

Non-Binary, Gender-Fluid and/or Two-Spirit

Gender not listed

1970s

12. In the 1970s how many Principal Investigators of each gender were involved in the project? A Principal Investigator on the project is defined as someone who actively participates in at least two of the following activities: seeking and administering core project funding, supervising student and/or postdoctoral projects, finalizing/approving hiring decisions, participates in project design, and/or holds a faculty position at a research institution.

Man

Woman

Non-Binary, Gender-Fluid and/or Two-Spirit

Gender not listed

13. In the 1970s how many Postdoctoral Researchers or Research Fellows of each gender were involved in the project? A Postdoctoral Researcher/Research Fellow on the project is defined as someone who has completed their PhD and has been hired by a Principal Investigator to conduct research pertaining to the project with a fixed end date.

Man

Woman

Non-Binary, Gender-Fluid and/or Two-Spirit

Gender not listed

14. In the 1970s how many graduate students of each gender were involved in the project? A graduate student on the project is defined as someone who is conducting their research as part of a graduate degree (i.e. Master’s or Doctorate).

Man

Woman

Non-Binary, Gender-Fluid and/or Two-Spirit

Gender not listed

15. In the 1970s how many other employees (such as field technicians and lab managers) of each gender were involved in the project? An employee of the project is defined as someone who holds a paid position to actively participate in project research, but does not fit into any of the above categories.

Man

Woman

Non-Binary, Gender-Fluid and/or Two-Spirit

Gender not listed

UNIVERSITY OF SASKATCHEWAN

Copy of Researcher gender in long-term individual-based ecological studies [Principal Investigator Survey]

1980s

16. In the 1980s how many Principal Investigators of each gender were involved in the project? A Principal Investigator on the project is defined as someone who actively participates in at least two of the following activities: seeking and administering core project funding, supervising student and/or postdoctoral projects, finalizing/approving hiring decisions, participates in project design, and/or holds a faculty position at a research institution.

Man

Woman

Non-Binary, Gender-Fluid and/or Two-Spirit

Gender not listed

17. In the 1980s how many Postdoctoral Researchers or Research Fellows of each gender were involved in the project? A Postdoctoral Researcher/Research Fellow on the project is defined as someone who has completed their PhD and has been hired by a Principal Investigator to conduct research pertaining to the project with a fixed end date.

Man	
Woman	
Non-Binary, Gender-Fluid and/or Two-Spirit	
Gender not listed	

18. In the 1980s how many graduate students of each gender were involved in the project? A graduate student on the project is defined as someone who is conducting their research as part of a graduate degree (i.e. Master's or Doctorate).

Man	
Woman	
Non-Binary, Gender-Fluid and/or Two-Spirit	
Gender not listed	

19. In the 1980s how many other employees (such as field technicians and lab managers) of each gender were involved in the project? An employee of the project is defined as someone who holds a paid position to actively participate in project research, but does not fit into any of the above categories.

Man	
Woman	
Non-Binary, Gender-Fluid and/or Two-Spirit	
Gender not listed	

UNIVERSITY OF SASKATCHEWAN

Copy of Researcher gender in long-term individual-based ecological studies [Principal Investigator Survey]

1990s

20. In the 1990s how many Principal Investigators of each gender were involved in the project? A Principal Investigator on the project is defined as someone who actively participates in at least two of the following activities: seeking and administering core project funding, supervising student and/or postdoctoral projects, finalizing/approving hiring decisions, participates in project design, and/or holds a faculty position at a research institution.

Man

Woman

Non-Binary, Gender-Fluid and/or Two-Spirit

Gender not listed

21. In the 1990s how many Postdoctoral Researchers or Research Fellows of each gender were involved in the project? A Postdoctoral Researcher/Research Fellow on the project is defined as someone who has completed their PhD and has been hired by a Principal Investigator to conduct research pertaining to the project with a fixed end date.

Man

Woman

Non-Binary, Gender-Fluid and/or Two-Spirit

Gender not listed

22. In the 1990s how many graduate students of each gender were involved in the project? A graduate student on the project is defined as someone who is conducting their research as part of a graduate degree (i.e. Master’s or Doctorate).

Man

Woman

Non-Binary, Gender-Fluid and/or Two-Spirit

Gender not listed

23. In the 1990s how many other employees (such as field technicians and lab managers) of each gender were involved in the project? An employee of the project is defined as someone who holds a paid position to actively participate in project research, but does not fit into any of the above categories.

Man	
Woman	
Non-Binary, Gender-Fluid and/or Two-Spirit	
Gender not listed	

UNIVERSITY OF SASKATCHEWAN

Copy of Researcher gender in long-term individual-based ecological studies [Principal Investigator Survey]

2000s

24. From 2000 to 2009 how many Principal Investigators of each gender were involved in the project? A Principal Investigator on the project is defined as someone who actively participates in at least two of the following activities: seeking and administering core project funding, supervising student and/or postdoctoral projects, finalizing/approving hiring decisions, participates in project design, and/or holds a faculty position at a research institution.

Man	
Woman	
Non-Binary, Gender-Fluid and/or Two-Spirit	
Gender not listed	

25. From 2000 to 2009 how many Postdoctoral Researchers or Research Fellows of each gender were involved in the project? A Postdoctoral Researcher/Research Fellow on the project is defined as someone who has completed their PhD and has been hired by a Principal Investigator to conduct research pertaining to the project with a fixed end date.

Man

Woman

Non-Binary, Gender-Fluid and/or Two-Spirit

Gender not listed

26. From 2000 to 2009 how many graduate students of each gender were involved in the project? A graduate student on the project is defined as someone who is conducting their research as part of a graduate degree (i.e. Master's or Doctorate).

Man

Woman

Non-Binary, Gender-Fluid and/or Two-Spirit

Gender not listed

27. From 2000 to 2009 how many other employees (such as field technicians and lab managers) of each gender were involved in the project? An employee of the project is defined as someone who holds a paid position to actively participate in project research, but does not fit into any of the above categories.

Man

Woman

Non-Binary, Gender-Fluid and/or Two-Spirit

Gender not listed

UNIVERSITY OF SASKATCHEWAN

Copy of Researcher gender in long-term individual-based ecological studies [Principal Investigator Survey]

2010s

28. From 2010-2019 how many Principal Investigators of each gender were involved in the project? A Principal Investigator on the project is defined as someone who actively participates in at least two of the following activities: seeking and administering core project funding, supervising student and/or postdoctoral projects, finalizing/approving hiring decisions, participates in project design, and/or holds a faculty position at a research institution.

Man

Woman

Non-Binary, Gender-Fluid and/or Two-Spirit

Gender not listed

29. From 2010-2019 how many Postdoctoral Researchers or Research Fellows of each gender were involved in the project? A Postdoctoral Researcher/Research Fellow on the project is defined as someone who has completed their PhD and has been hired by a Principal Investigator to conduct research pertaining to the project with a fixed end date.

Man

Woman

Non-Binary, Gender-Fluid and/or Two-Spirit

Gender not listed

30. From 2010-2019 how many graduate students of each gender were involved in the project? A graduate student on the project is defined as someone who is conducting their research as part of a graduate degree (i.e. Master’s or Doctorate).

Man

Woman

Non-Binary, Gender-Fluid and/or Two-Spirit

Gender not listed

31. From 2010-2019 how many other employees (such as field technicians and lab managers) of each gender were involved in the project? An employee of the project is defined as someone who holds a paid position to actively participate in project research, but does not fit into any of the above categories.

Man

Woman

Non-Binary, Gender-Fluid and/or Two-Spirit

Gender not listed

UNIVERSITY OF SASKATCHEWAN

Copy of Researcher gender in long-term individual-based ecological studies [Principal Investigator Survey]

2020s

32. Since 2020 how many Principal Investigators of each gender were involved in the project? A Principal Investigator on the project is defined as someone who actively participates in at least two of the following activities: seeking and administering core project funding, supervising student and/or postdoctoral projects, finalizing/approving hiring decisions, participates in project design, and/or holds a faculty position at a research institution.

Man

Woman

Non-Binary, Gender-Fluid and/or Two-Spirit

Gender not listed

33. Since 2020 how many Postdoctoral Researchers or Research Fellows of each gender were involved in the project? A Postdoctoral Researcher/Research Fellow on the project is defined as someone who has completed their PhD and has been hired by a Principal Investigator to conduct research pertaining to the project with a fixed end date.

Man

Woman

Non-Binary, Gender-Fluid and/or Two-Spirit

Gender not listed

34. Since 2020 how many graduate students of each gender were involved in the project? A graduate student on the project is defined as someone who is conducting their research as part of a graduate degree (i.e. Master’s or Doctorate).

Man

Woman

Non-Binary, Gender-Fluid and/or Two-Spirit

Gender not listed

35. Since 2020 how many other employees (such as field technicians and lab managers) of each gender were involved in the project? An employee of the project is defined as someone who holds a paid position to actively participate in project research, but does not fit into any of the above categories.

Man

Woman

Non-Binary, Gender-Fluid and/or Two-Spirit

Gender not listed

UNIVERSITY OF SASKATCHEWAN

Copy of Researcher gender in long-term individual-based ecological studies [Principal Investigator Survey]

Last Q

36. Is there any other information you would like to include? If you have suggestions for other projects you feel should be involved in this work that were not listed, please indicate them below
