# Supplementary material for: Experiences and Gender Demographics of Researchers in Long‐Term Individual‐Based Study Systems
Source: Ecol Evol. 2026 Nov 6;16(11):e74577. doi: 10.1002/ece3.74577 (PMC13743406; doi:10.1002/ece3.74577)
Supplement: Supplementary file 1 [file ECE3-16-e74577-s001.zip › ece374577-sup-0003-supinfo03.pdf]

Supplementary Information:

Table S1. The effect of role and year and their interaction on long-term individual-based ecology project participation by gender. The dependent variable is the number of men and people of marginalized genders bound as a single object. The base factor of role is employee.

Variables	Estimate	z value	P value
Intercept	0.20 ± 0.37	0.53	0.60
Role_Grad	0.58 ± 0.52	1.13	0.26
Role_PDF	0.56 ± 0.85	0.65	0.51
Role_PI	1.61 ± 0.57	2.81	<0.01
Year	-0.11 ± 0.09	-1.21	0.23
Role_Grad:Year	-0.28 ± 0.14	-2.00	0.05
Role_PDF:Year	-0.21 ± 0.23	-0.91	0.36
Role_PI:Year	-0.29 ± 0.16	-1.87	0.06

Table S2. The effect of role and year and their interaction on long-term individual-based ecology project participation by gender. The dependent variable is the number of men and women bound as a single object. The base factor of role is employee.

Variables	Estimate	z value	P value
Intercept	0.18 ± 0.37	0.47	0.64
Role_Grad	0.64 ± 0.52	1.24	0.22
Role_PDF	0.53 ± 0.86	0.62	0.54
Role_PI	1.62 ± 0.57	2.83	<0.01
Year	-0.10 ± 0.09	-1.10	0.27
Role_Grad:Year	-0.30 ± 0.14	-2.10	0.04
Role_PDF:Year	-0.20 ± 0.23	-0.88	0.38
Role_PI:Year	-0.30 ± 0.16	-1.93	0.06

Table S3. The effect of PI gender, role, year, and their interactions on gender of participants on long-term individual-based ecology projects. The dependent variable is the number of men and people of marginalized genders bound as a single object. The base factor of role is employee.

Variables	Estimate	z value	P value
Intercept	0.12 ± 0.58	0.21	0.84
Role_Grad	-0.97 ± 0.26	-3.71	<0.01
Role_PDF	-0.96 ± 0.43	-2.23	0.03
PI_ratio	0.75 ± 0.82	0.91	0.36
Year	-0.05 ± 0.14	-0.35	0.73
Role_Grad:PI_ratio	1.27 ± 0.42	3.01	<0.01
Role_PDF:PI_ratio	1.66 ± 0.67	2.46	0.01
Year:PI_ratio	-0.36 ± 0.21	-1.76	0.08

Table S4. The effect of PI gender, role, year, and their interactions on gender of participants on long-term individual-based ecology projects. The dependent variable is the number of men and women bound as a single object. The base factor of role is employee.

Variables	Estimate	z value	P value
Intercept	0.14 ± 0.58	0.24	0.81
Role_Grad	-0.96 ± 0.26	-3.66	<0.01
Role_PDF	-0.95 ± 0.43	-2.20	0.03
PI_ratio	0.71 ± 0.82	0.86	0.39
Year	-0.05 ± 0.14	-0.38	0.70
Role_Grad:PI_ratio	1.27 ± 0.42	3.00	<0.01
Role_PDF:PI_ratio	1.63 ± 0.67	2.42	0.02
Year:PI_ratio	-0.35 ± 0.21	-1.69	0.09

Table S5. Model summary of the effect of gender, role, and their interaction on coauthorship publication rates of participants of long-term research projects. The model compares men (base factor) to all marginalized genders grouped together. The base factor of role is graduate student.

Variables (Count Model)	Estimate	z value	P value
Intercept	-0.61 ± 0.24	-2.52	0.01
Marginalized_Genders	0.41 ± 0.26	1.55	0.12
Role_Other	-0.38 ± 0.58	-0.66	0.51
Role_PDF	0.6 ± 0.29	2.08	0.04
Role_PI	1.7 ± 0.25	6.81	<0.01
Marginalized_Genders:Role_Other	0.05 ± 0.62	0.08	0.94
Marginalized_Genders:Role_PDF	-0.06 ± 0.35	-0.18	0.85
Marginalized_Genders:Role_PI	-0.95 ± 0.28	-3.42	<0.01
Variables (Zero Model)	Estimate	z value	P value
Intercept	0.48 ± 0.79	0.608	0.54
Marginalized_Genders	0.06 ± 0.93	0.061	0.95
Role_Other	-1.17 ± 1.35	-0.869	0.39
Role_PDF	0.14 ± 1.33	0.101	0.92
Role_PI	-0.74 ± 1.47	-0.502	0.62
Marginalized_Genders:Role_Other	-0.12 ± 1.55	-0.074	0.94
Marginalized_Genders:Role_PDF	-0.08 ± 1.79	-0.047	0.96
Marginalized_Genders:Role_PI	15.06 ± 2550.90	0.006	1.00

Table S6. Model summary of the effect of gender, role, and their interaction on coauthorship publication rates of participants of long-term research projects. The model compares men (base factor) to women. The base factor of role is graduate student.

Variables (Count Model)	Estimate	z value	P value
Intercept	-0.61 ± 0.24	-2.52	0.01
Women	0.44 ± 0.26	1.67	0.10
Role_Other	-0.38 ± 0.58	-0.66	0.51
Role_PDF	0.6 ± 0.29	2.08	0.04
Role_PI	1.7 ± 0.25	6.81	0.00
Women:Role_Other	0.07 ± 0.62	0.11	0.91
Women:Role_PDF	-0.08 ± 0.35	-0.22	0.83
Women:Role_PI	-0.98 ± 0.28	-3.53	<0.01
Variables (Zero Model)	Estimate	z value	P value
Intercept	0.48 ± 0.79	0.61	0.54
Women	-0.02 ± 0.93	-0.02	0.99
Role_Other	-1.17 ± 1.35	-0.87	0.39
Role_PDF	0.14 ± 1.33	0.10	0.92
Role_PI	-0.74 ± 1.47	-0.50	0.62
Women:Role_Other	-0.08 ± 1.58	-0.05	0.96
Women:Role_PDF	-0.17 ± 1.81	-0.09	0.93
Women:Role_PI	15.13 ± 2550.90	0.01	1.00

S7. Model summary of the effect of gender, role, and their interaction on first author publication rates of participants of long-term research projects. The model compares men (base factor) to all marginalized genders grouped together. The base factor of role is graduate student.

Variables (Count Model)	Estimate	z value	P value
Intercept	-0.92 ± 0.32	-2.84	0.00
Marginalized_Genders	0.22 ± 0.36	0.61	0.54
Role_Other	-0.34 ± 0.72	-0.48	0.63
Role_PDF	0.46 ± 0.39	1.18	0.24
Role_PI	0.70 ± 0.35	2.01	0.04
Marginalized_Genders:Role_Other	-0.59 ± 0.84	-0.71	0.48
Marginalized_Genders:Role_PDF	-0.14 ± 0.49	-0.29	0.77
Marginalized_Genders:Role_PI	-0.44 ± 0.4	-1.09	0.28
Variables (Zero Model)	Estimate	z value	P value
Intercept	-0.05 ± 0.68	-0.08	0.94
Marginalized_Genders	-0.48 ± 0.78	-0.61	0.54
Role_Other	-1.99 ± 1.45	-1.37	0.17
Role_PDF	-0.77 ± 1.00	-0.77	0.44
Role_PI	-2.16 ± 1.08	-2.00	0.05
Marginalized_Genders:Role_Other	1.42 ± 1.61	0.88	0.38
Marginalized_Genders:Role_PDF	1.89 ± 1.53	1.24	0.22
Marginalized_Genders Role_PI	0.65 ± 1.45	0.45	0.65

S8. Model summary of the effect of gender, role, and their interaction on first author publication rates of participants of long-term research projects. The model compares men (base factor) to women. The base factor of role is graduate student.

Variables (Count Model)	Estimate	z value	P value
Intercept	-0.92 ± 0.32	-2.84	0.00
Women	0.23 ± 0.36	0.63	0.53
Role_Other	-0.34 ± 0.72	-0.48	0.63
Role_PDF	0.46 ± 0.39	1.18	0.24
Role_PI	0.7 ± 0.35	2.01	0.04
Women:Role_Other	-0.55 ± 0.84	-0.65	0.51
Women:Role_PDF	-0.09 ± 0.49	-0.18	0.86
Women:Role_PI	-0.45 ± 0.41	-1.10	0.27
Variables (Zero Model)	Estimate	z value	P value
Intercept	-0.05 ± 0.68	-0.08	0.94
Women	-0.43 ± 0.79	-0.54	0.59
Role_Other	-1.99 ± 1.45	-1.37	0.17
Role_PDF	-0.77 ± 1	-0.77	0.44
Role_PI	-2.16 ± 1.08	-2.00	0.05
Women:Role_Other	1.26 ± 1.64	0.77	0.44
Women:Role_PDF	1.68 ± 1.55	1.09	0.28
Women:Role_PI	0.6 ± 1.46	0.41	0.68

S9. Model summary of the effect of gender on last author publication rates of participants of long-term individual-based research projects. The model compares men (base factor) to all marginalized genders grouped together.

Variables (Count Model)	Estimate	z value	P value
Intercept	0.65 ± 0.07	9.71	<0.01
Marginalized_Genders	-1.47 ± 0.16	-9.34	<0.01
Variables (Zero Model)	Estimate	z value	P value
Intercept	-2.99 ± 0.46	-6.57	<0.01
Marginalized_Genders	-0.53 ± 0.60	-0.88	0.38

S10. Model summary of the effect of gender on last author publication rates of participants of long-term individual-based research projects. The model compares men (base factor) to women.

Variables (Count Model)	Estimate	z value	P value
Intercept	0.65 ± 0.07	9.71	<0.01
Women	-1.44 ± 0.16	-9.18	<0.01
Variables (Zero Model)	Estimate	z value	P value
Intercept	-2.99 ± 0.46	-6.57	<0.01
Women	-0.63 ± 0.62	-1.02	0.31

S11. Model summary of the effects of gender on coauthorship publication rates of participants of long-term research project with one high productive outlier removed. The model compares men (base factor) to marginalized genders. The base factor of role is graduate student.

Variables (Count Model)	Estimate	z value	P value
Intercept	-0.61 ± 0.24	-2.52	0.01
Marginalized_Gender	0.41 ± 0.26	1.55	0.12
Role2Other	-0.38 ± 0.58	-0.66	0.51
Role2PDF	0.6 ± 0.29	2.08	0.04
Role2PI	1.29 ± 0.25	5.06	0.00
Marginalized_Gender:RoleOther	0.05 ± 0.62	0.08	0.94
Marginalized_Gender:RolePDF	-0.06 ± 0.35	-0.18	0.85
Marginalized_Gender:RolePI	-0.54 ± 0.28	-1.92	0.05

Variables (Zero Model)	Estimate	z value	P value
Intercept	0.48 ± 0.79	0.61	0.54
Marginalized_Gender	0.06 ± 0.93	0.06	0.95
Role2Other	-1.17 ± 1.35	-0.87	0.39
Role2PDF	0.14 ± 1.33	0.10	0.92
Role2PI	-0.83 ± 1.5	-0.56	0.58
Marginalized_Gender:RoleOther	-0.12 ± 1.55	-0.07	0.94
Marginalized_Gender:RolePDF	-0.08 ± 1.79	-0.05	0.96
Marginalized_Gender:RolePI	15.15 ± 2550.90	0.01	1.00

S12. Model summary of the effects of gender on last author publication rates of participants of long-term research project with one high productive outlier removed. The model compares men (base factor) to marginalized genders.

Variables (Count Model)	Estimate	z value	P value
Intercept	0.03 ± 0.1	0.31	0.76
Marginalized_Genders	-0.85 ± 0.17	-4.84	<0.01

Variables (Zero Model)	Estimate	z value	P value
Intercept	-3.1 ± 0.48	-6.46	<0.01
Marginalized_Genders	-0.43 ± 0.62	-0.68	0.49
